# Comparability of Point-of-Care versus Central Laboratory Hemoglobin Determination in Emergency Patients at a Supra-Maximal Care Hospital

**DOI:** 10.1371/journal.pone.0166521

**Published:** 2016-11-23

**Authors:** Ramona C. Dolscheid-Pommerich, Sarah Dolscheid, Daniel Grigutsch, Birgit Stoffel-Wagner, Ingo Graeff

**Affiliations:** 1 Department of Clinical Chemistry and Clinical Pharmacology, University Hospital Bonn, Sigmund- Freud- Str. 25, 53127 Bonn, Germany; 2 Department of Rehabilitation and Special Education, University of Cologne, Herbert-Lewin-Str. 10, 50931 Köln, Germany; 3 Department of Anesthesiology and Intensive Care Medicine, University Hospital Bonn, Sigmund- Freud- Str. 25, 53127 Bonn, Germany; 4 Emergency Department, University Hospital Bonn, Sigmund- Freud- Str. 25, 53127 Bonn, Germany; Virgen Macarena University Hospital, School of Medicine, University of Seville, SPAIN

## Abstract

Fulfilling the requirements of point-of-care testing (POCT) training regarding proper execution of measurements and compliance with internal and external quality control specifications is a great challenge. Our aim was to compare the values of the highly critical parameter hemoglobin (Hb) determined with POCT devices and central laboratory analyzer in the highly vulnerable setting of an emergency department in a supra maximal care hospital to assess the quality of POCT performance. In 2548 patients, Hb measurements using POCT devices (POCT-Hb) were compared with Hb measurements performed at the central laboratory (Hb-ZL). Additionally, sub collectives (WHO anemia classification, patients with Hb <8 g/dl and suprageriatric patients (age >85y.) were analyzed. Overall, the correlation between POCT-Hb and Hb-ZL was highly significant (*r* = 0.96, *p*<0.001). Mean difference was -0.44g/dl. POCT-Hb values tended to be higher than Hb-ZL values (*t*(2547) = 36.1, *p*<0.001). Standard deviation of the differences was 0.62 g/dl. Only in 26 patients (1%), absolute differences >2.5g/dl occurred. McNemar´s test revealed significant differences regarding anemia diagnosis according to WHO definition for male, female and total patients (♂ *p*<0.001; ♀ *p*<0.001, total *p*<0.001). Hb-ZL resulted significantly more often in anemia diagnosis. In samples with Hb<8g/dl, McNemar´s test yielded no significant difference (*p* = 0.169). In suprageriatric patients, McNemar´s test revealed significant differences regarding anemia diagnosis according to WHO definition in male, female and total patients (♂ *p*<0.01; ♀ *p* = 0.002, total *p*<0.001). The difference between Hb-ZL and POCT-Hb with Hb<8g/dl was not statistically significant (<8g/dl, *p* = 1.000). Overall, we found a highly significant correlation between the analyzed hemoglobin concentration measurement methods, i.e. POCT devices and at the central laboratory. The results confirm the successful implementation of the presented POCT concept. Nevertheless some limitations could be identified in anemic patients stressing the importance of carefully examining clinically implausible results.

## Introduction

Implementation and importance of point-of-care testing (POCT) has steadily increased in recent years among established practitioners as well as in hospitals. The main feature of POCT is its access to diagnosis right at the patient’s bedside, which allows for integration into treatment processes, e.g. emergency care, with immediate therapeutic and diagnostic consequences. In Germany, the guideline of the German Medical Association (Richtlinie der Bundesärztekammer, RiliBÄK) stipulates requirements for quality control in medical laboratories, which also include POCT measurements [1]. The advantages of POCT include low sample volumes, less invasive sample collection, and the elimination of long transport periods and sample preparation procedures. The main disadvantage is the potentially higher costs. Moreover, comparability of POCT measurement results with the results from other methods is not always warranted. Regarding patient safety, correct sample identification and subsequent documentation are mandatory requirements. Also, POCT can mean considerable extra work for non-laboratory trained staff members. Therefore, the efficiency of POCT training regarding proper execution of measurements and compliance with internal and external quality control requirements is critical [2]. These issues highlight the challenges in developing and introducing a legally binding and realizable POCT concept [3].

### Importance of the study

In line with legal requirements (RiliBÄK), a comprehensive POCT concept was developed and implemented at the University Clinics Bonn, Germany (UKB) to enable effective use of the advantages of POCT in this supra maximal care hospital. Accurate and rapidly accessible results that allow immediate therapeutic and diagnostic consequences are an essential requirement in the treatment of critically ill patients [4]. Bedside measurements can be optimally integrated into emergency algorithms, e.g. trauma room treatment. At UKB, POCT measurements are performed as part of emergency care by a member of the interdisciplinary emergency center team. Shifting of this core capability from the central laboratory to an “operational” area can result in measurement deviations [5]. Also, findings obtained at the patient’s bedside are produced under time pressure with immediate therapeutic consequences. Therefore, a POCT concept has to ensure a reliably efficient bedside diagnosis.

### Aim of the study

Aim of the study was to compare the values of the highly critical parameter hemoglobin (Hb) determined with POCT devices and central laboratory analyzer in the highly vulnerable setting of an emergency department in a supra maximal care hospital to assess the quality of POCT performance. For the study design, the interdisciplinary emergency center was deliberately chosen as a highly vulnerable area with a large caseload and high stress level. Economic observations, such as a cost benefit analysis, were not the focus of the present study.

## Materials and Methods

### Setting

This is a single-center retrospective observational study performed at the central laboratory and the interdisciplinary emergency center (Interdisziplinäres Notfallzentrum, INZ) of the University Clinics Bonn, Germany (UKB). UKB is a supra maximal care hospital with 1250 beds and approx. 2600 POCT users. With full research and teaching responsibilities, all medical disciplines are represented at the University Clinics Bonn. Currently, 550 000 POCT measurements and 400 000 laboratory measurements are carried out annually. INZ is certified as a level-1 trauma center (approx. 600 trauma room patients) and is an interdisciplinary section of the cardiac arrest center with approx. 100 cardiopulmonary resuscitations annually.

Over the course of 24 hours, nine nurses work on weekdays and 11 nurses on the weekend, in a mix of specialist nurses, employees with additional qualifications and paramedics. All medical staff members and all nursing staff were trained in POCT diagnosis. All sample measurements carried out by the 24 care employees were included in the analysis. During the evaluation period, there was no change in observational conditions, e.g. number of staff or workflow; POCT training also remained unchanged. At the central laboratory, specialists in laboratory medicine, residents and laboratory technicians are in attendance 24 hours/day. In addition, from Monday to Friday, two POCT coordinators are responsible for internal and external POCT quality control and training of POCT users. There were no changes in the workflow and staff conditions at the central laboratory during the observation period. Blood sampling for POCT and central laboratory measurements were done concurrently from the same sampling point. Absolute values of the surrogate marker hemoglobin and specific cut-off values with differing clinical relevance were analyzed.

### POCT concept at UKB

The POCT concept at UKB was established with different areas of responsibilities. Table 1 shows the responsibility levels and detailed areas of responsibility.

**Table 1:** Responsibility levels of POCT at UKB.

Responsibility level	Detailed areas of responsibility
Medical director of the central laboratory and POCT coordination	
	Internal and external quality control
	Current device status (middleware)
	User administration
	Management of devices
	Management of back-up devices
	Checking for outliers
	Measurements with back-up devices
	Purchase ordering of reagents
	Execution of ring trials
	Hotline
	Performance statistics
	User training: blood gas analysis, blood glucose, coagulation
User	
	Measurement of patient sample
	Measurement of control sample
	Consumables: Refilling, emptying, ordering
Manufacturers	
	Maintenance and repair of the devices

Table 1 shows the responsibility levels and detailed areas of responsibility. The POCT concept distinguishes three responsibility levels: the medical director of the central laboratory with the POCT coordination, users and manufacturers. Detailed areas of responsibility are presented.

The training sessions are carried out by the POCT coordinators of the central laboratory or by instructors authorized by the manufacturers, on a weekly basis and upon individual appointment. The one-hour long training sessions consist of preanalytics, measurement, trouble shooting etc.; specific initial and follow-up training sessions, troubleshooting and intensive training sessions are provided for staff in charge of devices. As stipulated by RiliBÄK, activation for measurement with the respective device is only effected after successful completion of the training. In case of misconduct, the user will be cautioned and blocked from measuring. Internal quality control measurements are performed automatically during each shift (every eight hours) in three different concentration areas.

In addition, the clinical director of UKB has appointed a POCT commission, which is tasked with creating a capable team for constructive and trustworthy supervision of the comprehensive area of POCT diagnosis. Commission members are appointed in line with RiliBÄK stipulations and include, inter alia, the medical director of the central laboratory, POCT coordinators, users, IT managers and purchasing managers.

### Central laboratory measurements

The reference methods results for the Hb value at the central laboratory (Hb-ZL) were established with Sysmex XN9000™ and Sysmex XN1000™ (Sysmex, Norderstedt, Germany). Hb concentration is photometrically measured with the SLS hemoglobin method (SLS = Sodium-Lauryl-Sulfate). Examination material for the central laboratory analysis consisted of venous EDTA whole blood. In all samples, analysis was performed within 15 minutes of arrival at the central laboratory.

### POCT measurements

At INZ, POCT-Hb diagnosis is done with System Rapidlap™1265 (Siemens Healthcare Diagnostics, Eschborn, Germany). For blood gas analysis, arterial, venous, capillary and mixed venous heparinized whole blood samples can be used. Co-oximetry allows measurement of blood oxygenation as well as total Hb concentration. Examination material for the blood gas analysis was heparinized venous whole blood.

In line with RiliBÄK, both methods used at UKB are subject to internal and external quality control under the responsibility of the medical director of the central laboratory. Accordingly, the stipulated external quality control requires participation in ring trials where results from hemoglobin measurements must not exceed a particular reference method value (cyanhemoglobin method) (Hb: maximum 6% deviation from target value). These conditions have been met regularly for Hb-ZL and POCT-Hb. In fact, for POCT-Hb as well as Hb-ZL, all results were significantly below the maximum 6% deviation from the target value as stipulated by the guidelines (66 ring trials: POCT-Hb deviation from target value: median 1.72% (5th/95th percentile 0.0%/5.59%); 59 ring trials: Hb-ZL deviation from target value: median 0.78% (5th/95th percentile 0.0%/2.66%)). Thus, the obtained ring trial values reveal an exceptionally high degree of accuracy for both measurement methods.

### Patient collective / collected data

The collective included data from 2548 patients, who received emergency care treatment during the first quarter of 2015 with POCT-Hb measurements and Hb-ZL measurements. Data were collected from the central laboratory information system (SWISSLAB II, Roche, Berlin Germany). For anemia diagnosis at an emergency department of a supra maximal care hospital, Hb measurements using POCT devices were compared with Hb measurements performed at the central laboratory as part of routine diagnosis with SLS detection. Blood sampling for POCT and central laboratory measurements were done concurrently. The following steps were performed:

1. Collating of differences between POCT-Hb and Hb-ZL measurements (as absolute value of Hb in mg/dl, hereafter referred to as absolute difference)
2. Analysis of the collective according to WHO anemia classification
3. Analysis of the collective with Hb <8 g/dl.
4. Analysis of the suprageriatric collective (patient age ≥85 years)

### Statistics

Data were statistically analyzed (Microsoft Excel, Version 2007; IBM SPSS Statistics, Version 20). *P* <0.05 was considered statistically significant. Variables (age and absolute difference of Hb value) were described with mean value, min, max, Hb values with mean value ± SD (standard deviation) and male-to-female ratio. Next, correlations were calculated and t-tests were used to compare the measurement results. However, since correlation coefficients do not provide sufficient results for a method comparison, the data were additionally summarized in a Bland-Altman plot. Here, the difference of the Hb values–as analyzed by the two methods–(Hb-ZL—POCT-Hb) is calculated for each patient and plotted against the mean value of both measurements ((Hb-ZL + POCT-Hb)/2). The limits of agreement of the Bland-Altman plot (i.e. the interval within which 95% of differences between measurements by the two methods are expected to lie) were described. Analysis of the collective was done by using cross tables and significance was determined according to McNemar.

### Ethics

According to information obtained from the local ethics commission (Ethikkommission an der Medizinischen Fakultät der Rheinischen Friedrich-Wilhelms-Universität Bonn, Chairman K. Racké, MD, PhD, Professor, University Bonn) retrospective analysis of data obtained during routine treatment and diagnosis does not require consultation by the ethics commission pursuant to §15 of the medical professional code. All collected clinical data evaluated in this study were fully anonymized before analysis. Therefore, according to prior agreement with the local ethics committee and the data protection officer appointed by the University Clinics Bonn, verbal or written informed consent was not obtained. As stipulated in article six of the German Data Protection Act (https://recht.nrw.de/lmi/owa/br_text_anzeigen?v_id=10000000000000000495#), the physician may use existing patient data for retrospective analyses without explicitly asking for the consent of patients. The study design is consistent with the Declaration of Helsinki.

## Results

**Fig 1:**
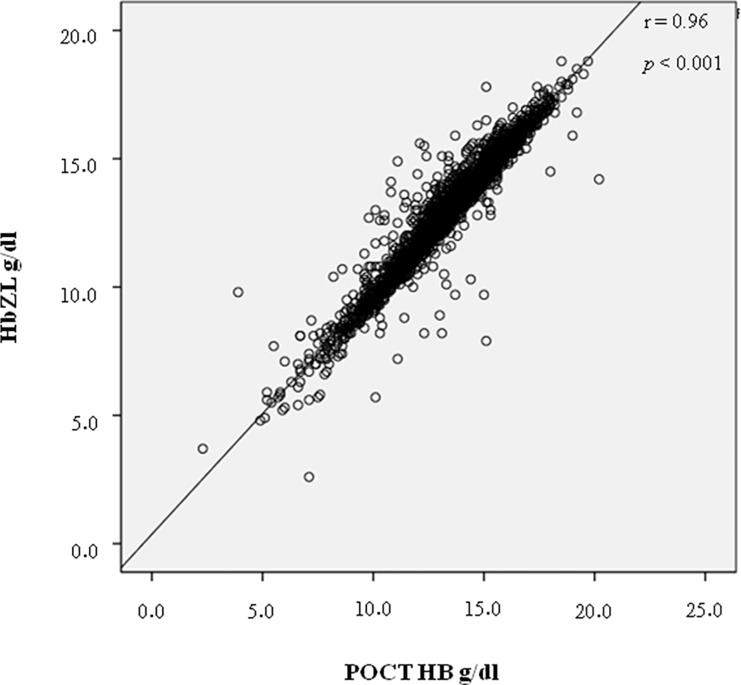
Correlation between the two measurement methods POCT-Hb and HB-ZL. Fig 1 shows the correlation between parameters POCT-Hb (g/dl) measured with POCT devices (Co-oximetry) and Hb-ZL (g/dl) measured with the SDS hemoglobin method.

**Fig 2:**
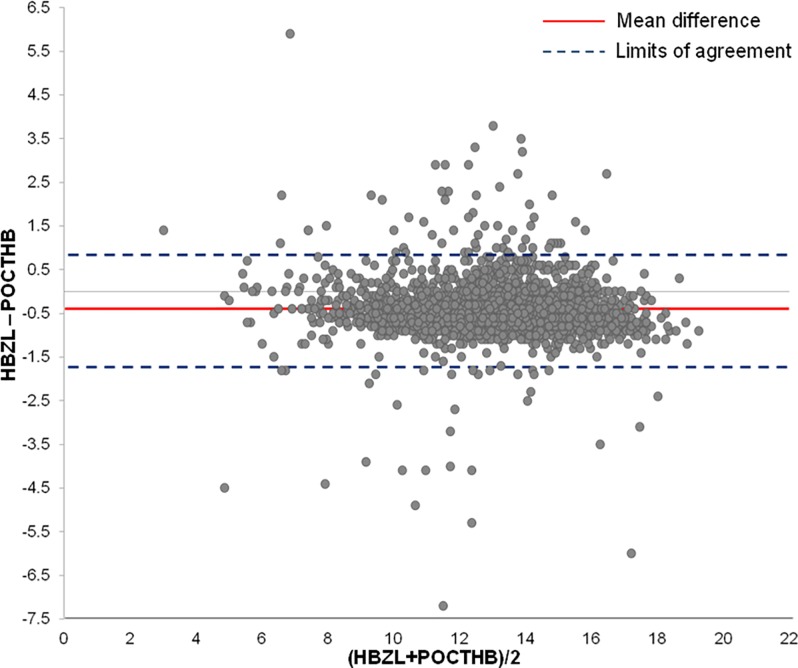
Bland-Altman plot showing the difference of the HB measurements. Fig 2 depicts the difference of the Hb values (Hb-ZL and POCT-Hb) calculated for each patient and plotted against the mean value of both measurements ((Hb-ZL + POCT-Hb)/2). The red line shows the mean difference of the measurement values (-0.44 g/dl). The dashed blue lines represent the limits of agreement within which 95% of differences between measurements by the two methods are expected to lie (-1.66 g/dl and +0.77 g/dl).

Average age of the 2548 analyzed patients was 56 years (range 7–99 years); data of two emergency patients were excluded due to inconclusive identity. Male-to-female ratio was 1363–1185 (53.5% male, 46.5% female). The absolute difference between POCT-Hb and Hb-ZL measurements yielded a mean value of 0.6 g/dl (range 0.0–7.2g/dl). Differences and descriptive statistics are shown in Tables 2 and 3. Hb-ZL mean value was 13.06 g/dl (SD 2.23), while POCT-Hb mean value was 13.51 g/dl (SD 2.28). The correlation between both parameters (POCT-Hb, Hb-ZL) was highly significant (*r* = 0.96, *p*< 0.001) (Fig 1). The mean difference of the measurement values was -0.44 g/dl (see Bland-Altman plot, Fig 2). On average, POCT-Hb values tended to be higher than Hb-ZL values (*t*(2547) = 36.1, *p*< 0.001). The standard deviation of the differences was 0.62 g/dl. Accordingly, the limits of agreement of the Bland-Altman plot range from -1.66 g/dl to +0.77 g/dl. There were a number of outliers, which are systematically analyzed below.

**Table 2:** Absolute difference between POCT-Hb and Hb-ZL measurements.

Absolute Difference	Samples (%)
0.0	89 (3.5)
0.1–0.5	1148 (44.9)
0.6–1.0	1157 (45.4)
1.1–1.5	94 (3.7)
1.6–2.0	21 (0.8)
2.1–2.5	13 (0.5)
>2.5	26 (1.0)

Table 2 shows the absolute and percentage samples grouped in 0.4 g/dl steps based on absolute differences (deviations up to 0.1% in percentage distribution are due to rounding).

**Table 3:** Descriptive statistics of the collective.

Age (years)	Number	Min abs diff.	Max abs. diff.	Mean diff. (SD)
<30	372	0.0	2.4	0.6 (0.32)
30–64	1157	0.0	7.2	0.6 (0.52)
65–84	827	0.0	5.9	0.6 (0.5)
>85	190	0.0	3.2	0.5 (0.42)

Table 3 shows the descriptive statistics with mean values ± SD (standard deviation), min and max according to age groups (abs diff. = absolute difference).

### Extreme outliers

In 26 patients, absolute differences >2.5 of measured Hb values occurred, indicating substantial differences between the two types of measurements. In this sub collective, male to female ratio was 11 to 15, with a median age of 59 years (range 30–85 years). Mean Hb measurements were 10.4 g/dl for Hb-ZL (range 2.6–17.8 g/dl) and 12.4 g/dl for POCT-Hb (range 3.9–20.2 g/dl). Four Hb-ZL values were flagged with the following comments: lipemic, hemolytic, while six POCT-Hb values were flagged with the comments: interfering substances, temperature corr., warning “check result”.

### WHO anemia

Table 4 shows the cross tables in anemic patients according to WHO classification (male <13g/dl, female <12g/dl) in the whole collective and separated by gender. The McNemar test revealed significant differences for male, female and whole patient collectives regarding anemia diagnosis when comparing POCT-Hb vs. Hb-ZL (♂ *p*<0.001; ♀ *p*<0.001, total *p*<0.001). Hb-ZL resulted significantly more often in an anemia diagnosis than POCT-Hb.

**Table 4:** Cross tables WHO classification.

**Male patients**				
	POCT-Hb in (%)	POCT-Hb out (%)		sens. 83.9%
				spec. 97.6%
Hb-ZL in (%)	380 (94.5)	73 (7.6)	453	
Hb-ZL out (%)	22 (5.5)	888 (92.4)	910	
	402 (100)	961 (100)	1363	
**Female patients**				
	POCT-Hb in (%)	POCT-Hb out (%)		sens. 76.5%
				spec. 97.7%
Hb-ZL in (%)	277 (93.6)	85 (9.6)	362	
Hb-ZL out (%)	19 (6.4)	804 (90.4)	823	
	296 (100)	889 (100)	1185	
**Whole patient collective**				
	POCT-Hb in (%)	POCT-Hb out (%)		sens. 80.6%
				spec. 97.6%
Hb-ZL in (%)	657 (94.1)	158 (8.5)	815	
Hb-ZL out (%)	41 (5.9)	1692 (91.5)	1733	
	698 (100)	1850 (100)	2548	

Table 4 shows the cross tables with sensitivity and specificity for POCT measurement in anemic patients according to WHO classification (male <13g/dl, female <12g/dl) in the whole collective and separately by gender. “In” indicates that patients are classified as anemic by the respective method (POCT, ZL), whereas “out” indicates that patients fall outside of the anemia cut-off according to the respective method.

### Anemia <8g/dl

Table 5 displays the cross table with a clinically relevant Hb value of <8g/dl. Here, in 42/2548 samples with Hb <8g/dl, the McNemar test yielded no significant difference between Hb-ZL and POCT-Hb (*p* = 0.169).

**Table 5:** Cross table Hb <8g/dl.

Hb <8g/dl				
	POCT-Hb in (%)	POCT-Hb out (%)		sens. 71.2%
				spec. 99.6%
Hb-ZL in (%)	42 (82.4)	17 (0.7)	59	
Hb-ZL out (%)	9 (17.6)	2480 (99.3)	2489	
	51 (100)	2497 (100)	2548	

Table 5 shows the cross table with sensitivity and specificity for POCT measurement in anemic patients with Hb <8g/dl. “In” indicates that patients are classified as anemic by the respective method (POCT, ZL), whereas “out” indicates that patients fall outside of the anemia cut-off according to the respective method.

### Sub collective suprageriatric patients

Table 3 (see page 10) shows the mean values ± SD (standard deviation), min and max of absolute differences grouped by age. Anemia sub analysis according to WHO and with Hb <8g/dl in suprageriatric patients is shown in Table 6 for male, female and whole patient collectives. In the collective of suprageriatric patients, the McNemar test revealed significant differences between Hb-ZL and POCT-Hb in male, female and whole patient collectives according to WHO classification (♂ *p*<0.008; ♀ *p* = 0.002, total *p*<0.001). The difference between Hb-ZL and POCT-Hb with Hb <8g/dl was not statistically significant (<8g/dl *p* = 1.000).

**Table 6:** Cross tables in suprageriatric patients.

**Male patients**				
	POCT-Hb in (%)	POCT-Hb out (%)		sens. 82.2%
				spec. 100%
Hb-ZL in (%)	37 (100)	8 (26.7)	45	
Hb-ZL out (%)	0 (0)	22 (73.3)	22	
	37 (100)	30 (100)	67	
**Female patients**				
	POCT-Hb in (%)	POCT-Hb out (%)		sens. 70.6%
				spec. 97.2%
Hb-ZL in (%)	36 (94.7)	15 (17.6)	51	
Hb-ZL out (%)	2 (5.3)	70 (82.4)	72	
	38 (100)	85 (100)	123	
**Whole patient collective**				
	POCT-Hb in (%)	POCT-Hb out (%)		sens. 76.0%
				spec. 97.9%
Hb-ZL in (%)	73 (97.3)	23 (20.0)	96	
Hb-ZL out (%)	2 (2.7)	92 (80.0)	94	
	75 (100)	115 (100)	190	
**Hb <8g/dl**				
POCT-Hb	POCT-Hb in (%)	POCT-Hb out (%)		sens. 83.3%
				spec. 99.5%
Hb-ZL in (%)	5 (83.3)	1 (0.5)	6	
Hb-ZL out (%)	1 (16.7)	183 (99.5)	184	
	6 (100)	184 (100)	190	

Table 6 shows the cross tables with sensitivity and specificity for POCT measurement according to WHO classification (male <13g/dl, female <12g/dl) in the whole suprageriatric (age >85 years) collective and separately by gender and for Hb <8g/dl. “In” indicates that patients are classified as anemic by the respective method (POCT, ZL), whereas “out” indicates that patients fall outside of the anemia cut-off according to the respective method.

## Discussion

At the University Clinics Bonn, a supra maximal care hospital, a POCT concept was developed and established to avail of the advantages of POCT effectively and in compliance with the law. In the present study, it was evaluated whether the POCT implementation did in fact result in the expected quality and efficiency, since diagnostic and therapeutic effects of POCT are considered to be of crucial importance. For the first time, the success of a POCT concept was verified in a highly vulnerable setting using the surrogate parameter hemoglobin. Main finding of this study is that the analytical accuracy of the established POCT concept at the UKB emergency center INZ meets all legal requirements regarding diagnosis quality and thus complies with therapeutic demands.

Studies in other institutions comparing laboratory measurements and POCT measurements of lactate, white blood cell count (WBC) and C-reactive protein (CRP) reported satisfactory, but also significantly divergent results [6,7]. In a Finnish pediatric emergency center, Ivaska et al. showed that WBC and CRP measured with a POCT device had sufficient analytical accuracy under local circumstances [6].

As a valid Hb measurement is a basic prerequisite in the first assessment, diagnosis and potentially therapy in an emergency patient, we opted for hemoglobin as a surrogate marker to evaluate the quality of the POCT concept at UKB. While in a previous study comparing POCT-Hb measurement in postoperative critical-ill patients with perioperative Hb-ZL measurements, a good consistency of the correlation coefficients was shown, minor systematic deviations were found when comparing the measurement systems [8]. A further study also compared Hb measurements. However, this was done in a small collective and results cannot be compared to the emergency centers of supra maximal hospitals [9].

The identified highly significant correlation between the two measurement methods (Hb-ZL and POCT-Hb) and the results from the Bland-Altman plot clearly attest to the feasibility of the established POCT concept. The results of the Bland-Altman plot indicate that in 95% of the cases, Hb-ZL measurements yield values that are up to -1.66 g/dl lower and up to 0.77 g/dl higher than POCT-Hb values [10,11]. Despite some deviations regarding the two types of measurements, the limits of agreement of the Bland-Altman plot indicate that the difference between POCT and Hb-ZL is comparatively small. However, some significant measurement deviations did occur. When examining these deviations more closely, it was found that only in 154 patients (i.e. 6% of the data) the difference exceeded >1g/dl. These findings demonstrate a good conformity between both measurement methods, which prooves the validity of the implemented POCT concept.

However, when looking at clinically relevant subgroups, even smaller differences between the two methods may have an impact. Internal UKB investigations have shown that transfusion is generally initiated at an Hb level of <8g/dl. Therefore, this cut-off level was considered to be clinically relevant in this study. In 17 cases with a Hb-ZL measurement level of <8g/dl, no transfusion would have been carried out if therapy decisions had been based on the POCT-Hb level, which was above the cut-off level. Since a similar difference regarding diagnosis of anemia was also found in male, female and total suprageriatric patients, this collective was separately evaluated. In order to detect possible weaknesses in the present POCT concept, outlier evaluation is mandatory. In some cases, we identified various interfering substances. However, in extreme situations, the enormous time pressure at the emergency center may lead to preanalytical errors, which are mainly due to comorbidities, difficulties with vein access and exsiccosis. The hematology analyzer at the central laboratory analyzes samples by overhead mixing to ensure sufficient and standardized mixing of the sample, whereas the POCT analysis at INZ requires sufficient manual mixing of the BGA vial. Clearly, this is not always done correctly and long enough. For example, in some cases, clot formation in the vials resulted in incorrect measurement values. Despite the fact that the BGA device had flagged the Hb value, the error report was ignored by the users and the incorrect value was applied. Therefore, proper training of POCT users is crucial to ensure that flagged values are recognized and incorrect measurement results are dismissed. As a consequence of the outlier evaluation, the areas of responsibilities of the POCT coordinators now include follow-up training and on-site troubleshooting, which are now provided individually and when problems arise.

With steadily decreasing resources and increasing costs through diagnosis, a scientific confirmation of the benefits of POCT in the decision making process in patient care has not been sufficiently examined to date [12]. Nevertheless, in many central emergency centers, POCT is the first step in the standardized emergency process in combination with a triage system. The triage system involves adequate categorization of priority levels immediately after patient admission at the emergency center [13]. As a consequence of mounting cost pressures, more and more in-hospital laboratories are outsourced resulting in an increase in POCT diagnosis. Therefore, studies on the comparability of POCT vs. laboratory results with an existing and lawful POCT concept are particularly important. Many hospitals not only measure blood gas analysis, but also clinical chemistry parameters with POCT. The quality of the results, however, can be problematic. Studies e.g. on troponin in the ED and on coagulation and renal function prior to interventional radiology and invasive cardiology have shown POCT diagnosis to be not as reliable and sensitive as central laboratory diagnosis [14, 15]. Also, pre-analysis plays a decisive role not only in the measurement of Hb, but of all parameters. Studies based in ICUs have identified a sound quality management as an essential component in order to achieve valid results [16]. Time to analysis is another critical factor. Studies on urine samples have shown that results differed even after a short period of time, e.g. if samples are transported to an external laboratory [17].

At INZ, POCT-Hb measurement is a standard procedure in patients with nosebleeds and parallel intake of anticoagulants immediately upon admission. The Hb value is used in the categorization of the priority level and can influence the next steps in the emergency treatment. Additionally, POCT diagnosis is essential for the clinical assessment of a patient, e.g. during primary survey in the trauma room [18]. Especially in critically ill patients, e.g. in acute emergencies or polytrauma, rapid access to laboratory values is crucial. Every second can be important for the patient outcome. Different scoring systems, such as the Trauma Associated Severe Hemorrhage (TASH) score used to predict mass transfusion, refer inter alia to the Hb value [19]. While rapid accessibility to results is a decisive advantage of POCT, the validity of the obtained measurement values has to be guaranteed [20]. During implementation as well as continuous quality control of POCT diagnosis in patient care, risks and benefits must be identified and minimized or maximized accordingly [21]. At UKB, all POCT quality control measures are the responsibility of the medical director of the central laboratory and thus by law equivalent to the quality control measures at the central laboratory. If a POCT device fails to pass the internal quality control, it is automatically disabled for the failed parameter and will only be released after successfully passing a follow-up control. The knowledge obtained from POCT error classifications has been included in our concept of training, follow-up training and troubleshooting [22].

### Limitations of the study

Preanalytical errors can simulate critical sample values in Hb-ZL and in POCT-Hb. Lipemic samples can also result in false high values in both methods. Cold agglutinins can macroscopically result in clotting. As the UKB central laboratory is located one floor above the UKB emergency center and both are connected with a pneumatic dispatch system, the critical period to analyze Hb (Hb measurement within four hours) could be adhered to in all cases in the present study.

## Conclusions

Our comparison of POCT versus central laboratory hemoglobin determination in emergency patients at a supra-maximal care hospital showed a highly significant correlation between the two analyzed hemoglobin concentration measurement methods, using POCT devices and the SLS hemoglobin method. Therefore, our study confirms the successful and RiliBÄK conform implementation of the shown POCT concept at UKB. The example of a highly vulnerable area such as the emergency center at UKB was used to demonstrate the successful implementation of a POCT concept in a supra maximal care hospital. Thus, results can be transferred to less vulnerable areas. A continuous quality control is of paramount importance. In Germany, this is based on adherence to the legally binding German Medical Association guidelines in medical laboratories (RiliBÄK), stipulating not only continuous internal and external quality control measures but also clearly defined areas of responsibility and ongoing user training [23]. With the chosen surrogate marker hemoglobin, some limitations could be identified in anemic patients stressing the importance of questioning clinically implausible results. In these cases, consultation with the central laboratory is necessary to decide on further diagnostic steps.

## Supporting Information

S1 File Performance evaluation. Performance evaluation of the central laboratory measurement method.
(DOC)

S1 Table Raw data. Raw data including age, sex, HB-Zl and POCT-Hb.
(PDF)
