# Supplementary material for: Comparability of Point-of-Care versus Central Laboratory Hemoglobin Determination in Emergency Patients at a Supra-Maximal Care Hospital
Source: PLoS One. 2016 Nov 23;11(11):e0166521. doi: 10.1371/journal.pone.0166521 (PMC5120806; doi:10.1371/journal.pone.0166521)
Supplement: S1 File — Performance evaluation of the central laboratory measurement method. (DOC) [file pone.0166521.s001.doc]

For our Sysmex XN analyzer, our intra-assay coefficients of variation are: 0.45 % (n=20; mean: 16.2 g/dl), 0.6 % (n=20; mean: 6.4 g/dl) and 0.55 % (n=20; mean: 12.3 g/dl) and our interassay coefficients of variation are 1.61 % & (n=30; mean: 6.25 g/dl ), 1.05 % (n=30; mean:12.3 g/dl) and 1.19% (n=30; mean:16.59 g/dl ) respectively.
