# Supplementary material for: Comparability of Point-of-Care versus Central Laboratory Hemoglobin Determination in Emergency Patients at a Supra-Maximal Care Hospital
Source: PLoS One. 2016 Nov 23;11(11):e0166521. doi: 10.1371/journal.pone.0166521 (PMC5120806; doi:10.1371/journal.pone.0166521)
Supplement: S1 Table — Raw data including age, sex, HB-Zl and POCT-Hb. (PDF) [file pone.0166521.s002.pdf]

AGE	SEX	HB-ZL	Result Hb-ZL	POCT-HB	Result POCT-HB
82	M	HB	11.2	P_THB	11.2
34	M	HB	6.3	P_THB	6.3
71	W	HB	10.8	P_THB	10.8
63	W	HB	12.6	P_THB	12.6
75	M	HB	13.2	P_THB	13.2
57	M	HB	14.2	P_THB	14.2
48	M	HB	14.5	P_THB	14.5
55	M	HB	14.9	P_THB	14.9
82	M	HB	14.0	P_THB	14.0
75	M	HB	14.1	P_THB	14.1
90	M	HB	5.7	P_THB	5.7
60	M	HB	13.0	P_THB	13.0
49	M	HB	14.1	P_THB	14.1
90	W	HB	10.3	P_THB	10.3
84	W	HB	12.1	P_THB	12.1
66	W	HB	7.8	P_THB	7.8
90	W	HB	12.2	P_THB	12.2
72	M	HB	12.0	P_THB	12.0
92	W	HB	12.7	P_THB	12.7
78	W	HB	12.4	P_THB	12.4
8	M	HB	13.5	P_THB	13.5
75	M	HB	15.4	P_THB	15.4
75	W	HB	16.9	P_THB	16.9
77	M	HB	14.2	P_THB	14.2
25	M	HB	15.1	P_THB	15.1
41	M	HB	16.0	P_THB	16.0
37	W	HB	15.2	P_THB	15.2
73	M	HB	14.2	P_THB	14.2
63	M	HB	8.7	P_THB	8.7
71	M	HB	12.1	P_THB	12.1
17	W	HB	15.3	P_THB	15.3
85	W	HB	11.0	P_THB	11.0
89	M	HB	9.6	P_THB	9.6
21	M	HB	14.6	P_THB	14.6
66	M	HB	14.9	P_THB	14.9
25	W	HB	14.2	P_THB	14.2
72	M	HB	12.8	P_THB	12.8
67	M	HB	12.8	P_THB	12.8
77	W	HB	14.3	P_THB	14.3
90	W	HB	15.6	P_THB	15.6
50	M	HB	14.9	P_THB	14.9
49	M	HB	13.3	P_THB	13.3
49	M	HB	14.0	P_THB	14.0
87	W	HB	10.1	P_THB	10.1
45	M	HB	15.7	P_THB	15.7
76	W	HB	12.5	P_THB	12.5
62	M	HB	13.7	P_THB	13.7
48	M	HB	15.4	P_THB	15.4
89	W	HB	7.1	P_THB	7.1
39	W	HB	13.6	P_THB	13.6
88	M	HB	6.7	P_THB	6.7
73	M	HB	15.4	P_THB	15.4
93	W	HB	17.5	P_THB	17.5
61	M	HB	16.5	P_THB	16.5
76	M	HB	13.4	P_THB	13.4

17	W	HB	14.3	P_THB	14.3
73	M	HB	15.3	P_THB	15.3
76	W	HB	11.0	P_THB	11.0
54	M	HB	16.7	P_THB	16.7
19	W	HB	14.5	P_THB	14.5
74	M	HB	9.0	P_THB	9.0
67	M	HB	11.2	P_THB	11.2
54	W	HB	9.1	P_THB	9.1
71	W	HB	14.3	P_THB	14.3
28	M	HB	15.3	P_THB	15.3
72	W	HB	15.0	P_THB	15.0
79	M	HB	12.3	P_THB	12.3
81	M	HB	13.6	P_THB	13.6
77	M	HB	13.8	P_THB	13.8
76	M	HB	8.7	P_THB	8.7
66	M	HB	16.9	P_THB	16.9
86	M	HB	11.3	P_THB	11.3
57	W	HB	12.9	P_THB	12.9
64	W	HB	5.8	P_THB	5.8
57	M	HB	14.5	P_THB	14.5
41	W	HB	13.4	P_THB	13.4
79	W	HB	13.4	P_THB	13.4
45	W	HB	13.7	P_THB	13.7
62	M	HB	11.0	P_THB	11.0
93	W	HB	12.5	P_THB	12.5
66	W	HB	11.9	P_THB	11.9
39	W	HB	16.4	P_THB	16.4
76	M	HB	10.7	P_THB	10.7
71	M	HB	13.3	P_THB	13.3
56	M	HB	13.3	P_THB	13.3
79	W	HB	10.8	P_THB	10.8
59	M	HB	13.6	P_THB	13.6
73	M	HB	14.9	P_THB	14.9
69	W	HB	10.3	P_THB	10.3
63	M	HB	17.2	P_THB	17.3
70	M	HB	15.2	P_THB	15.1
75	W	HB	13.3	P_THB	13.4
71	M	HB	12.1	P_THB	12.2
68	M	HB	15.1	P_THB	15.2
83	M	HB	14.2	P_THB	14.1
30	W	HB	14.1	P_THB	14.2
86	W	HB	8.4	P_THB	8.3
64	W	HB	10.9	P_THB	10.8
69	W	HB	10.8	P_THB	10.9
52	M	HB	9.3	P_THB	9.4
86	M	HB	11.3	P_THB	11.4
87	M	HB	12.9	P_THB	12.8
52	M	HB	14.8	P_THB	14.9
50	M	HB	14.3	P_THB	14.4
63	M	HB	11.2	P_THB	11.1
85	W	HB	11.8	P_THB	11.9
83	M	HB	13.1	P_THB	13.2
81	W	HB	10.3	P_THB	10.4
64	W	HB	10.8	P_THB	10.9
19	M	HB	15.1	P_THB	15.2
77	M	HB	13.8	P_THB	13.9

78	W	HB	15.3	P_THB	15.4
86	W	HB	15.1	P_THB	15.2
54	M	HB	12.1	P_THB	12.2
64	M	HB	14.1	P_THB	14.2
86	W	HB	10.8	P_THB	10.9
63	M	HB	9.1	P_THB	9.2
80	M	HB	11.2	P_THB	11.1
39	M	HB	11.2	P_THB	11.1
80	W	HB	12.3	P_THB	12.4
35	M	HB	15.1	P_THB	15.2
88	W	HB	12.4	P_THB	12.3
45	M	HB	14.8	P_THB	14.9
43	M	HB	13.9	P_THB	13.8
50	M	HB	15.8	P_THB	15.9
73	M	HB	11.8	P_THB	11.9
63	M	HB	14.1	P_THB	14.2
44	W	HB	9.6	P_THB	9.7
82	M	HB	12.7	P_THB	12.6
62	M	HB	12.3	P_THB	12.4
18	W	HB	14.1	P_THB	14.2
76	M	HB	9.7	P_THB	9.6
74	W	HB	14.8	P_THB	14.9
31	W	HB	11.1	P_THB	11.2
75	M	HB	13.1	P_THB	13.2
47	M	HB	15.9	P_THB	15.8
53	M	HB	15.6	P_THB	15.7
77	W	HB	12.2	P_THB	12.1
35	M	HB	15.7	P_THB	15.6
31	M	HB	14.7	P_THB	14.6
79	M	HB	11.7	P_THB	11.6
72	M	HB	15.2	P_THB	15.1
67	W	HB	12.4	P_THB	12.3
76	M	HB	12.1	P_THB	12.2
17	W	HB	14.1	P_THB	14.2
86	M	HB	5.9	P_THB	5.8
79	W	HB	7.2	P_THB	7.1
61	M	HB	5.5	P_THB	5.4
58	M	HB	4.8	P_THB	4.9
67	M	HB	15.2	P_THB	15.3
92	W	HB	12.4	P_THB	12.5
78	M	HB	11.9	P_THB	12.0
25	W	HB	13.9	P_THB	14.0
60	M	HB	11.4	P_THB	11.5
19	W	HB	12.5	P_THB	12.6
91	M	HB	8.5	P_THB	8.6
85	W	HB	13.5	P_THB	13.4
97	W	HB	14.3	P_THB	14.2
93	M	HB	13.5	P_THB	13.4
40	M	HB	17.1	P_THB	17.2
50	W	HB	14.0	P_THB	13.9
56	W	HB	14.9	P_THB	15.0
74	M	HB	9.4	P_THB	9.5
44	M	HB	13.1	P_THB	13.0
58	M	HB	10.1	P_THB	10.0
87	W	HB	13.4	P_THB	13.5
54	W	HB	14.1	P_THB	14.0

77	M	HB	15.0	P_THB	15.1
53	M	HB	15.2	P_THB	15.3
66	M	HB	14.0	P_THB	13.9
27	W	HB	9.7	P_THB	9.8
71	M	HB	10.8	P_THB	10.7
81	M	HB	13.9	P_THB	14.0
77	M	HB	15.2	P_THB	15.3
77	M	HB	10.7	P_THB	10.8
90	W	HB	14.7	P_THB	14.8
80	W	HB	13.2	P_THB	13.3
33	W	HB	12.4	P_THB	12.5
58	W	HB	13.4	P_THB	13.5
27	W	HB	13.3	P_THB	13.2
52	M	HB	15.3	P_THB	15.2
59	M	HB	14.7	P_THB	14.8
75	M	HB	8.4	P_THB	8.5
68	M	HB	10.6	P_THB	10.5
67	M	HB	11.7	P_THB	11.8
72	M	HB	9.1	P_THB	9.0
24	M	HB	15.2	P_THB	15.3
30	M	HB	15.4	P_THB	15.5
46	M	HB	15.7	P_THB	15.8
77	M	HB	12.9	P_THB	13.0
78	M	HB	8.4	P_THB	8.5
68	M	HB	10.0	P_THB	9.9
64	W	HB	13.9	P_THB	14.0
41	W	HB	14.7	P_THB	14.8
53	M	HB	11.6	P_THB	11.5
25	W	HB	15.1	P_THB	15.0
73	W	HB	13.6	P_THB	13.5
59	M	HB	14.7	P_THB	14.8
46	M	HB	17.6	P_THB	17.7
28	W	HB	13.5	P_THB	13.6
88	W	HB	9.8	P_THB	9.7
80	W	HB	13.0	P_THB	13.1
76	M	HB	9.5	P_THB	9.6
26	W	HB	12.5	P_THB	12.6
58	M	HB	11.6	P_THB	11.5
89	M	HB	12.7	P_THB	12.8
18	W	HB	13.7	P_THB	13.8
58	M	HB	13.5	P_THB	13.6
70	M	HB	10.0	P_THB	9.9
39	W	HB	12.7	P_THB	12.8
25	M	HB	14.5	P_THB	14.4
61	M	HB	12.9	P_THB	13.0
79	W	HB	13.5	P_THB	13.6
78	M	HB	11.9	P_THB	12.0
48	M	HB	15.5	P_THB	15.6
16	M	HB	15.4	P_THB	15.5
81	W	HB	12.3	P_THB	12.2
60	M	HB	12.2	P_THB	12.3
39	M	HB	16.0	P_THB	16.1
89	M	HB	12.2	P_THB	12.3
26	W	HB	8.5	P_THB	8.6
46	M	HB	13.2	P_THB	13.3
77	W	HB	8.3	P_THB	8.2

56	M	HB	16.2	P_THB	16.1
55	M	HB	13.9	P_THB	14.0
64	M	HB	14.9	P_THB	15.0
23	M	HB	16.1	P_THB	16.2
19	W	HB	13.5	P_THB	13.6
61	M	HB	14.2	P_THB	14.3
28	W	HB	14.1	P_THB	14.0
44	M	HB	16.0	P_THB	15.9
69	M	HB	8.5	P_THB	8.6
80	M	HB	14.0	P_THB	13.9
30	M	HB	14.4	P_THB	14.5
41	W	HB	11.5	P_THB	11.6
59	M	HB	13.2	P_THB	13.3
55	W	HB	14.2	P_THB	14.3
83	W	HB	8.4	P_THB	8.5
73	M	HB	14.3	P_THB	14.2
63	M	HB	12.8	P_THB	12.7
63	M	HB	6.8	P_THB	6.7
72	M	HB	10.0	P_THB	10.1
84	W	HB	17.7	P_THB	17.9
50	M	HB	16.6	P_THB	16.8
26	M	HB	13.1	P_THB	13.3
40	M	HB	14.6	P_THB	14.8
20	M	HB	15.6	P_THB	15.8
53	M	HB	15.1	P_THB	15.3
50	M	HB	12.3	P_THB	12.5
22	M	HB	15.5	P_THB	15.3
81	M	HB	10.0	P_THB	9.8
52	W	HB	13.8	P_THB	14.0
92	W	HB	12.8	P_THB	12.6
78	M	HB	9.4	P_THB	9.2
60	M	HB	12.0	P_THB	12.2
71	W	HB	14.1	P_THB	14.3
21	W	HB	11.3	P_THB	11.5
75	M	HB	15.3	P_THB	15.5
80	W	HB	11.5	P_THB	11.7
74	W	HB	13.8	P_THB	14.0
77	W	HB	4.9	P_THB	5.1
47	W	HB	11.8	P_THB	12.0
74	M	HB	12.7	P_THB	12.9
54	M	HB	12.9	P_THB	12.7
16	M	HB	14.8	P_THB	15.0
91	W	HB	14.6	P_THB	14.8
84	M	HB	10.8	P_THB	11.0
73	M	HB	10.7	P_THB	10.5
78	M	HB	11.6	P_THB	11.8
81	M	HB	8.2	P_THB	8.4
63	M	HB	14.2	P_THB	14.4
52	M	HB	14.5	P_THB	14.7
75	M	HB	10.3	P_THB	10.5
34	W	HB	13.6	P_THB	13.8
52	W	HB	12.7	P_THB	12.9
34	W	HB	13.8	P_THB	14.0
64	M	HB	14.7	P_THB	14.5
67	M	HB	12.5	P_THB	12.3
27	W	HB	14.2	P_THB	14.0

59	M	HB	12.7	P_THB	12.5
64	W	HB	9.8	P_THB	10.0
75	W	HB	9.8	P_THB	10.0
79	M	HB	13.7	P_THB	13.9
81	W	HB	14.2	P_THB	14.4
27	W	HB	13.3	P_THB	13.5
59	M	HB	12.7	P_THB	12.9
45	M	HB	14.0	P_THB	14.2
38	W	HB	10.0	P_THB	10.2
72	M	HB	8.0	P_THB	7.8
89	M	HB	12.8	P_THB	12.6
94	W	HB	14.3	P_THB	14.5
77	M	HB	12.2	P_THB	12.0
45	M	HB	15.5	P_THB	15.7
50	W	HB	15.5	P_THB	15.3
27	M	HB	14.2	P_THB	14.4
80	M	HB	9.2	P_THB	9.0
63	M	HB	12.7	P_THB	12.9
76	W	HB	12.8	P_THB	13.0
82	W	HB	11.7	P_THB	11.9
54	W	HB	12.8	P_THB	12.6
95	W	HB	12.8	P_THB	12.6
57	W	HB	14.5	P_THB	14.7
24	M	HB	15.7	P_THB	15.9
46	W	HB	11.0	P_THB	11.2
66	M	HB	10.8	P_THB	11.0
26	M	HB	14.8	P_THB	15.0
22	W	HB	11.0	P_THB	10.8
81	M	HB	12.7	P_THB	12.9
35	M	HB	14.4	P_THB	14.2
60	M	HB	13.8	P_THB	14.0
39	W	HB	13.4	P_THB	13.2
79	M	HB	14.2	P_THB	14.4
58	W	HB	10.5	P_THB	10.7
89	M	HB	14.8	P_THB	15.0
48	M	HB	12.2	P_THB	12.0
54	M	HB	15.1	P_THB	15.3
88	M	HB	7.8	P_THB	8.0
78	M	HB	11.8	P_THB	12.0
89	M	HB	14.4	P_THB	14.2
58	M	HB	14.5	P_THB	14.7
61	W	HB	10.2	P_THB	10.4
74	M	HB	12.4	P_THB	12.2
58	M	HB	7.8	P_THB	8.0
27	W	HB	13.7	P_THB	13.9
41	W	HB	12.1	P_THB	12.3
36	W	HB	11.6	P_THB	11.8
77	M	HB	8.7	P_THB	8.9
26	W	HB	14.7	P_THB	14.9
77	M	HB	10.7	P_THB	10.9
60	W	HB	12.6	P_THB	12.8
55	M	HB	14.1	P_THB	14.3
26	W	HB	12.6	P_THB	12.8
79	W	HB	11.3	P_THB	11.1
37	W	HB	11.0	P_THB	11.2
78	W	HB	11.1	P_THB	11.3

21	W	HB	13.5	P_THB	13.7
47	M	HB	15.7	P_THB	15.5
46	W	HB	12.7	P_THB	12.5
73	W	HB	9.7	P_THB	9.9
34	M	HB	14.8	P_THB	15.0
45	M	HB	14.5	P_THB	14.3
78	M	HB	12.6	P_THB	12.8
19	W	HB	12.6	P_THB	12.8
82	W	HB	11.8	P_THB	12.0
50	W	HB	12.3	P_THB	12.1
59	M	HB	15.8	P_THB	16.0
85	W	HB	13.8	P_THB	13.6
61	W	HB	12.2	P_THB	12.0
39	W	HB	12.5	P_THB	12.7
72	W	HB	14.0	P_THB	14.2
50	W	HB	12.2	P_THB	12.4
74	M	HB	12.7	P_THB	12.5
65	M	HB	12.0	P_THB	12.2
88	W	HB	13.1	P_THB	13.3
82	M	HB	7.4	P_THB	7.6
34	W	HB	11.1	P_THB	11.3
73	W	HB	12.7	P_THB	12.5
28	W	HB	13.2	P_THB	13.4
72	M	HB	8.4	P_THB	8.2
79	M	HB	15.8	P_THB	16.0
22	W	HB	14.2	P_THB	14.4
33	M	HB	13.9	P_THB	13.7
71	W	HB	13.2	P_THB	13.4
67	M	HB	15.5	P_THB	15.7
39	M	HB	13.2	P_THB	13.4
28	M	HB	14.5	P_THB	14.7
57	W	HB	10.7	P_THB	10.9
93	W	HB	10.3	P_THB	10.5
43	W	HB	13.4	P_THB	13.2
38	M	HB	13.2	P_THB	13.4
42	M	HB	14.0	P_THB	14.2
46	M	HB	15.3	P_THB	15.1
72	M	HB	11.7	P_THB	11.9
73	W	HB	10.8	P_THB	10.6
49	M	HB	13.1	P_THB	13.3
28	M	HB	13.8	P_THB	14.0
28	M	HB	11.1	P_THB	11.3
86	W	HB	10.3	P_THB	10.5
76	M	HB	12.3	P_THB	12.1
58	M	HB	15.6	P_THB	15.8
59	W	HB	13.3	P_THB	13.5
52	W	HB	9.3	P_THB	9.5
31	W	HB	14.2	P_THB	14.4
39	W	HB	12.1	P_THB	12.3
75	W	HB	15.5	P_THB	15.7
46	M	HB	9.6	P_THB	9.8
71	M	HB	7.7	P_THB	7.9
52	W	HB	7.9	P_THB	8.1
52	W	HB	11.9	P_THB	12.1
67	W	HB	12.4	P_THB	12.6
34	W	HB	13.9	P_THB	14.1

53	M	HB	12.4	P_THB	12.6
83	W	HB	12.9	P_THB	13.1
58	M	HB	16.0	P_THB	16.2
46	M	HB	14.9	P_THB	15.1
14	W	HB	12.1	P_THB	11.9
28	M	HB	14.9	P_THB	15.1
77	M	HB	13.1	P_THB	12.9
30	M	HB	16.9	P_THB	17.1
70	M	HB	16.0	P_THB	16.2
70	W	HB	11.9	P_THB	12.1
79	M	HB	13.1	P_THB	12.9
47	M	HB	16.2	P_THB	16.0
23	M	HB	17.5	P_THB	17.7
61	M	HB	13.9	P_THB	14.1
44	M	HB	16.0	P_THB	16.2
57	W	HB	16.1	P_THB	15.9
20	W	HB	11.4	P_THB	11.6
65	M	HB	9.6	P_THB	9.4
24	W	HB	10.4	P_THB	10.6
76	M	HB	13.4	P_THB	13.6
72	M	HB	16.5	P_THB	16.7
26	M	HB	14.9	P_THB	15.1
65	M	HB	13.6	P_THB	13.4
37	W	HB	12.9	P_THB	13.1
34	M	HB	13.9	P_THB	14.1
84	W	HB	10.4	P_THB	10.6
47	W	HB	13.9	P_THB	14.1
46	M	HB	16.3	P_THB	16.5
75	M	HB	11.9	P_THB	12.1
72	M	HB	12.9	P_THB	13.1
29	M	HB	15.4	P_THB	15.6
29	W	HB	12.6	P_THB	12.4
49	M	HB	16.0	P_THB	16.2
50	M	HB	15.4	P_THB	15.6
22	W	HB	12.6	P_THB	12.4
81	W	HB	12.4	P_THB	12.6
26	W	HB	11.6	P_THB	11.4
71	W	HB	9.4	P_THB	9.6
77	M	HB	16.1	P_THB	15.9
54	W	HB	15.6	P_THB	15.4
93	W	HB	12.9	P_THB	12.6
78	M	HB	14.1	P_THB	14.4
45	M	HB	14.1	P_THB	14.4
85	W	HB	12.1	P_THB	12.4
70	M	HB	11.1	P_THB	11.4
89	W	HB	11.6	P_THB	11.9
60	M	HB	15.1	P_THB	15.4
73	M	HB	12.6	P_THB	12.9
26	W	HB	13.6	P_THB	13.9
71	W	HB	15.9	P_THB	15.6
75	W	HB	11.1	P_THB	11.4
77	M	HB	14.1	P_THB	14.4
48	W	HB	11.6	P_THB	11.9
59	W	HB	15.1	P_THB	15.4
50	W	HB	13.4	P_THB	13.1
74	M	HB	14.4	P_THB	14.1

39	M	HB	14.1	P_THB	14.4
68	W	HB	14.6	P_THB	14.9
83	M	HB	8.1	P_THB	8.4
82	M	HB	12.6	P_THB	12.9
84	M	HB	9.9	P_THB	9.6
38	W	HB	14.6	P_THB	14.9
48	W	HB	11.1	P_THB	11.4
46	M	HB	9.1	P_THB	9.4
83	M	HB	13.1	P_THB	13.4
64	W	HB	10.6	P_THB	10.9
62	M	HB	16.2	P_THB	16.5
16	M	HB	16.7	P_THB	17.0
55	M	HB	17.0	P_THB	17.3
81	M	HB	15.6	P_THB	15.9
26	W	HB	11.6	P_THB	11.9
79	W	HB	11.9	P_THB	11.6
77	W	HB	12.1	P_THB	12.4
53	M	HB	10.1	P_THB	10.4
83	M	HB	10.1	P_THB	10.4
29	M	HB	15.1	P_THB	15.4
65	M	HB	11.6	P_THB	11.9
36	W	HB	13.6	P_THB	13.9
53	M	HB	14.1	P_THB	14.4
94	W	HB	12.1	P_THB	12.4
69	M	HB	13.4	P_THB	13.1
63	M	HB	8.6	P_THB	8.9
28	M	HB	15.9	P_THB	15.6
49	M	HB	14.6	P_THB	14.9
36	M	HB	14.1	P_THB	14.4
50	M	HB	12.1	P_THB	12.4
57	W	HB	13.1	P_THB	13.4
80	W	HB	10.6	P_THB	10.9
42	M	HB	14.6	P_THB	14.9
79	M	HB	18.8	P_THB	18.5
34	W	HB	13.9	P_THB	13.6
90	M	HB	8.2	P_THB	7.9
44	W	HB	11.7	P_THB	12.0
56	M	HB	15.1	P_THB	14.8
66	W	HB	15.7	P_THB	15.4
63	M	HB	13.1	P_THB	12.8
55	M	HB	14.3	P_THB	14.6
62	W	HB	9.9	P_THB	10.2
63	M	HB	14.9	P_THB	15.2
59	W	HB	12.3	P_THB	12.6
42	W	HB	13.5	P_THB	13.8
87	M	HB	11.7	P_THB	12.0
27	M	HB	15.6	P_THB	15.3
66	W	HB	14.5	P_THB	14.8
83	W	HB	10.5	P_THB	10.8
89	W	HB	11.7	P_THB	12.0
54	W	HB	11.5	P_THB	11.2
75	M	HB	15.2	P_THB	15.5
71	M	HB	14.3	P_THB	14.6
51	M	HB	12.8	P_THB	13.1
26	M	HB	14.9	P_THB	15.2
71	M	HB	15.4	P_THB	15.7

26	W	HB	15.0	P_THB	15.3
67	W	HB	11.0	P_THB	11.3
50	W	HB	11.3	P_THB	11.6
78	W	HB	12.0	P_THB	12.3
20	W	HB	13.1	P_THB	12.8
59	W	HB	13.9	P_THB	14.2
38	W	HB	12.0	P_THB	12.3
54	W	HB	14.8	P_THB	15.1
79	M	HB	13.4	P_THB	13.7
47	M	HB	12.8	P_THB	13.1
79	W	HB	12.5	P_THB	12.2
40	M	HB	14.8	P_THB	15.1
75	M	HB	12.8	P_THB	13.1
47	M	HB	9.2	P_THB	9.5
70	W	HB	10.4	P_THB	10.7
38	M	HB	15.6	P_THB	15.3
74	M	HB	13.2	P_THB	13.5
49	W	HB	13.5	P_THB	13.2
71	M	HB	12.0	P_THB	12.3
34	W	HB	13.7	P_THB	14.0
78	M	HB	9.0	P_THB	8.7
89	M	HB	12.3	P_THB	12.6
48	M	HB	10.0	P_THB	10.3
72	M	HB	8.4	P_THB	8.7
69	W	HB	13.7	P_THB	14.0
21	M	HB	12.2	P_THB	12.5
62	W	HB	14.2	P_THB	14.5
63	M	HB	13.9	P_THB	14.2
88	W	HB	14.7	P_THB	15.0
54	M	HB	14.9	P_THB	15.2
62	M	HB	8.8	P_THB	9.1
72	W	HB	12.7	P_THB	13.0
84	W	HB	12.5	P_THB	12.8
42	M	HB	13.2	P_THB	12.9
91	M	HB	11.4	P_THB	11.7
77	M	HB	8.8	P_THB	9.1
49	M	HB	13.8	P_THB	14.1
86	W	HB	12.7	P_THB	13.0
94	W	HB	9.3	P_THB	9.6
59	W	HB	13.7	P_THB	14.0
24	M	HB	14.7	P_THB	15.0
76	M	HB	13.8	P_THB	13.5
96	W	HB	15.1	P_THB	14.8
68	M	HB	13.9	P_THB	14.2
59	W	HB	10.8	P_THB	11.1
78	M	HB	9.4	P_THB	9.7
62	M	HB	15.7	P_THB	16.0
21	M	HB	15.7	P_THB	15.4
63	W	HB	11.2	P_THB	11.5
44	M	HB	15.5	P_THB	15.8
58	M	HB	14.4	P_THB	14.7
79	W	HB	13.0	P_THB	12.7
41	W	HB	13.9	P_THB	14.2
81	W	HB	11.7	P_THB	12.0
36	W	HB	13.9	P_THB	14.2
81	M	HB	14.2	P_THB	14.5

63	W	HB	13.8	P_THB	14.1
43	W	HB	14.4	P_THB	14.7
62	M	HB	11.8	P_THB	12.1
83	M	HB	14.4	P_THB	14.7
76	M	HB	13.4	P_THB	13.7
18	W	HB	11.8	P_THB	12.1
26	W	HB	15.7	P_THB	16.0
37	M	HB	15.5	P_THB	15.8
16	M	HB	13.0	P_THB	13.3
82	W	HB	11.5	P_THB	11.2
30	M	HB	15.8	P_THB	16.1
78	M	HB	10.7	P_THB	11.0
66	M	HB	7.6	P_THB	7.9
35	M	HB	15.4	P_THB	15.7
69	M	HB	14.3	P_THB	14.6
46	M	HB	13.9	P_THB	14.2
56	M	HB	11.7	P_THB	12.0
63	W	HB	14.4	P_THB	14.7
59	W	HB	12.9	P_THB	13.2
33	M	HB	14.3	P_THB	14.0
83	M	HB	11.3	P_THB	11.6
92	W	HB	8.4	P_THB	8.7
52	W	HB	11.7	P_THB	12.0
62	W	HB	15.7	P_THB	16.0
55	W	HB	9.9	P_THB	10.2
72	W	HB	14.7	P_THB	15.0
86	W	HB	12.4	P_THB	12.7
40	M	HB	14.5	P_THB	14.8
82	W	HB	11.3	P_THB	11.6
60	M	HB	13.9	P_THB	14.2
44	W	HB	12.0	P_THB	11.7
25	M	HB	15.8	P_THB	16.1
83	W	HB	14.4	P_THB	14.7
90	M	HB	14.8	P_THB	15.1
72	M	HB	13.9	P_THB	14.2
34	W	HB	14.0	P_THB	14.3
65	M	HB	11.0	P_THB	11.3
51	M	HB	8.8	P_THB	9.1
91	M	HB	15.8	P_THB	16.1
84	W	HB	12.9	P_THB	13.2
60	M	HB	15.0	P_THB	15.3
64	W	HB	14.3	P_THB	14.0
29	W	HB	14.3	P_THB	14.6
88	W	HB	10.9	P_THB	11.2
57	W	HB	13.5	P_THB	13.8
94	M	HB	10.5	P_THB	10.8
70	M	HB	10.5	P_THB	10.8
38	M	HB	14.3	P_THB	14.0
24	W	HB	13.8	P_THB	14.1
55	W	HB	13.9	P_THB	14.2
68	W	HB	12.3	P_THB	12.6
26	M	HB	14.0	P_THB	14.3
37	W	HB	12.8	P_THB	13.1
84	W	HB	11.0	P_THB	10.7
49	M	HB	13.3	P_THB	13.6
41	W	HB	14.3	P_THB	14.6

76	W	HB	12.2	P_THB	12.5
33	W	HB	14.3	P_THB	14.6
45	W	HB	13.2	P_THB	13.5
47	W	HB	9.0	P_THB	9.3
47	M	HB	13.8	P_THB	14.1
68	W	HB	13.0	P_THB	13.3
57	M	HB	13.3	P_THB	13.6
53	M	HB	9.5	P_THB	9.8
77	M	HB	14.0	P_THB	14.3
54	M	HB	9.1	P_THB	8.8
80	M	HB	12.2	P_THB	12.5
62	M	HB	14.8	P_THB	15.1
84	W	HB	12.7	P_THB	13.0
76	M	HB	15.0	P_THB	15.3
76	M	HB	11.2	P_THB	11.5
80	W	HB	13.4	P_THB	13.7
49	W	HB	11.8	P_THB	12.1
78	M	HB	12.8	P_THB	13.1
96	W	HB	11.7	P_THB	11.4
71	M	HB	11.5	P_THB	11.8
52	W	HB	13.0	P_THB	13.3
34	W	HB	14.7	P_THB	15.0
23	M	HB	14.7	P_THB	15.0
40	W	HB	12.8	P_THB	13.1
41	M	HB	13.9	P_THB	14.2
61	M	HB	14.9	P_THB	15.2
71	W	HB	7.4	P_THB	7.1
33	M	HB	9.8	P_THB	10.1
69	M	HB	14.2	P_THB	14.5
23	W	HB	13.5	P_THB	13.8
73	W	HB	7.8	P_THB	7.5
45	M	HB	16.4	P_THB	16.7
37	M	HB	15.9	P_THB	16.2
62	M	HB	15.9	P_THB	16.2
60	M	HB	16.4	P_THB	16.7
26	M	HB	16.6	P_THB	16.3
29	M	HB	16.3	P_THB	16.6
45	M	HB	16.4	P_THB	16.7
75	M	HB	14.6	P_THB	15.0
71	W	HB	13.5	P_THB	13.9
60	M	HB	15.5	P_THB	15.9
46	M	HB	15.3	P_THB	15.7
23	W	HB	14.5	P_THB	14.9
77	M	HB	14.8	P_THB	15.2
77	W	HB	12.7	P_THB	12.3
59	M	HB	16.5	P_THB	16.9
90	W	HB	11.0	P_THB	11.4
84	M	HB	16.2	P_THB	16.6
44	W	HB	12.0	P_THB	11.6
81	W	HB	13.4	P_THB	13.0
23	W	HB	13.3	P_THB	13.7
13	W	HB	12.8	P_THB	13.2
70	M	HB	11.0	P_THB	11.4
21	M	HB	15.6	P_THB	16.0
62	W	HB	14.5	P_THB	14.9
22	W	HB	11.8	P_THB	12.2

60	M	HB	14.0	P_THB	14.4
75	M	HB	13.3	P_THB	13.7
83	W	HB	11.3	P_THB	11.7
83	W	HB	11.1	P_THB	11.5
56	M	HB	13.3	P_THB	13.7
64	W	HB	12.4	P_THB	12.0
21	W	HB	14.1	P_THB	14.5
76	W	HB	11.0	P_THB	11.4
74	W	HB	12.1	P_THB	12.5
26	M	HB	15.0	P_THB	15.4
67	M	HB	14.0	P_THB	14.4
61	M	HB	13.1	P_THB	13.5
51	M	HB	16.2	P_THB	16.6
21	W	HB	12.8	P_THB	13.2
74	W	HB	13.8	P_THB	14.2
45	W	HB	11.1	P_THB	11.5
43	M	HB	14.5	P_THB	14.9
39	W	HB	12.9	P_THB	12.5
75	W	HB	11.8	P_THB	12.2
41	W	HB	10.8	P_THB	11.2
93	W	HB	10.5	P_THB	10.1
48	W	HB	14.1	P_THB	14.5
48	W	HB	13.0	P_THB	13.4
56	W	HB	14.6	P_THB	15.0
65	M	HB	8.8	P_THB	9.2
75	M	HB	10.6	P_THB	11.0
59	W	HB	13.6	P_THB	14.0
62	M	HB	11.1	P_THB	11.5
43	W	HB	12.5	P_THB	12.9
32	W	HB	12.8	P_THB	13.2
41	M	HB	15.5	P_THB	15.9
88	W	HB	10.5	P_THB	10.9
81	W	HB	15.1	P_THB	15.5
75	M	HB	9.6	P_THB	10.0
86	W	HB	13.1	P_THB	13.5
83	M	HB	13.1	P_THB	13.5
82	M	HB	15.5	P_THB	15.9
96	W	HB	14.6	P_THB	15.0
30	W	HB	15.0	P_THB	15.4
74	M	HB	15.9	P_THB	16.3
57	W	HB	13.4	P_THB	13.0
79	W	HB	10.0	P_THB	10.4
23	M	HB	15.0	P_THB	15.4
83	M	HB	6.3	P_THB	6.7
36	W	HB	13.0	P_THB	13.4
59	W	HB	15.0	P_THB	15.4
83	W	HB	12.0	P_THB	12.4
55	W	HB	14.0	P_THB	13.6
72	W	HB	14.0	P_THB	14.4
25	W	HB	14.3	P_THB	14.7
66	W	HB	13.8	P_THB	14.2
57	M	HB	16.9	P_THB	17.3
83	M	HB	13.5	P_THB	13.9
23	W	HB	11.0	P_THB	11.4
24	M	HB	15.5	P_THB	15.9
58	M	HB	11.1	P_THB	11.5

67	M	HB	10.8	P_THB	11.2
66	W	HB	11.6	P_THB	12.0
25	W	HB	10.3	P_THB	10.7
62	M	HB	9.0	P_THB	9.4
30	W	HB	13.3	P_THB	13.7
74	W	HB	14.0	P_THB	14.4
33	M	HB	16.4	P_THB	16.8
55	M	HB	14.5	P_THB	14.9
49	M	HB	17.1	P_THB	17.5
77	W	HB	11.8	P_THB	12.2
88	M	HB	17.8	P_THB	17.4
23	M	HB	15.9	P_THB	16.3
34	M	HB	13.6	P_THB	14.0
25	W	HB	10.8	P_THB	11.2
34	M	HB	9.5	P_THB	9.9
99	W	HB	11.1	P_THB	11.5
22	M	HB	15.1	P_THB	15.5
28	W	HB	10.0	P_THB	10.4
28	W	HB	10.0	P_THB	10.4
24	W	HB	14.0	P_THB	14.4
42	M	HB	14.6	P_THB	15.0
63	W	HB	12.9	P_THB	12.5
78	W	HB	15.9	P_THB	15.5
57	W	HB	13.8	P_THB	14.2
29	W	HB	13.1	P_THB	13.5
78	M	HB	14.1	P_THB	14.5
27	W	HB	14.5	P_THB	14.9
33	M	HB	13.6	P_THB	14.0
81	M	HB	11.8	P_THB	12.2
68	W	HB	14.0	P_THB	14.4
60	M	HB	14.1	P_THB	14.5
40	M	HB	15.6	P_THB	16.0
69	M	HB	12.6	P_THB	13.0
63	M	HB	15.9	P_THB	16.3
72	M	HB	8.5	P_THB	8.9
80	M	HB	16.1	P_THB	16.5
72	M	HB	8.9	P_THB	8.5
57	M	HB	13.5	P_THB	13.1
69	W	HB	15.0	P_THB	15.4
75	M	HB	11.8	P_THB	12.2
69	M	HB	12.6	P_THB	13.0
68	M	HB	13.6	P_THB	14.0
58	W	HB	13.6	P_THB	14.0
96	W	HB	13.4	P_THB	13.0
48	M	HB	13.8	P_THB	14.2
66	W	HB	14.1	P_THB	14.5
57	W	HB	14.3	P_THB	14.7
76	M	HB	11.5	P_THB	11.9
18	W	HB	11.3	P_THB	11.7
29	W	HB	14.1	P_THB	14.5
51	W	HB	15.1	P_THB	15.5
39	W	HB	11.1	P_THB	11.5
66	W	HB	11.8	P_THB	12.2
33	W	HB	10.6	P_THB	11.0
71	M	HB	14.3	P_THB	14.7
33	M	HB	14.0	P_THB	14.4

65	M	HB	14.1	P_THB	14.5
40	W	HB	14.1	P_THB	14.5
46	W	HB	12.3	P_THB	12.7
44	M	HB	9.1	P_THB	9.5
40	W	HB	12.6	P_THB	13.0
67	M	HB	8.5	P_THB	8.1
18	W	HB	14.6	P_THB	15.0
37	M	HB	16.0	P_THB	16.4
86	W	HB	12.3	P_THB	12.7
69	W	HB	13.1	P_THB	13.5
56	M	HB	12.5	P_THB	12.9
44	M	HB	14.8	P_THB	15.2
55	M	HB	10.5	P_THB	10.9
72	M	HB	11.0	P_THB	11.4
43	W	HB	13.6	P_THB	14.0
23	W	HB	7.0	P_THB	7.4
64	M	HB	5.6	P_THB	5.2
83	M	HB	7.0	P_THB	7.4
83	W	HB	7.0	P_THB	7.4
74	W	HB	7.0	P_THB	6.6
77	M	HB	7.4	P_THB	7.8
67	M	HB	7.2	P_THB	7.6
84	M	HB	6.7	P_THB	7.1
89	M	HB	12.7	P_THB	13.1
24	W	HB	10.4	P_THB	10.8
20	W	HB	13.9	P_THB	14.3
76	M	HB	15.7	P_THB	16.1
83	M	HB	11.4	P_THB	11.8
26	M	HB	14.9	P_THB	15.3
76	M	HB	15.7	P_THB	16.1
37	W	HB	12.9	P_THB	13.3
22	M	HB	15.7	P_THB	16.1
62	W	HB	14.4	P_THB	14.8
36	W	HB	13.7	P_THB	14.1
79	M	HB	12.2	P_THB	12.6
77	W	HB	11.7	P_THB	12.1
71	W	HB	12.9	P_THB	13.3
26	W	HB	14.9	P_THB	15.3
23	W	HB	13.2	P_THB	13.6
59	W	HB	13.2	P_THB	13.6
60	M	HB	15.4	P_THB	15.8
22	W	HB	10.7	P_THB	11.1
81	W	HB	12.2	P_THB	12.6
79	M	HB	11.7	P_THB	12.1
50	M	HB	15.8	P_THB	16.2
64	M	HB	14.7	P_THB	15.1
30	W	HB	13.2	P_THB	13.6
74	M	HB	10.2	P_THB	10.6
51	M	HB	15.2	P_THB	15.6
59	W	HB	13.7	P_THB	14.1
75	M	HB	10.1	P_THB	9.7
84	W	HB	10.7	P_THB	11.1
20	W	HB	12.7	P_THB	13.1
68	M	HB	10.9	P_THB	11.3
33	W	HB	14.9	P_THB	15.3
58	M	HB	11.8	P_THB	11.4

64	W	HB	11.9	P_THB	12.3
46	W	HB	14.7	P_THB	15.1
58	W	HB	9.2	P_THB	9.6
78	M	HB	13.7	P_THB	14.1
34	W	HB	14.2	P_THB	14.6
86	W	HB	14.2	P_THB	14.6
43	M	HB	14.4	P_THB	14.8
87	W	HB	13.9	P_THB	14.3
40	W	HB	13.2	P_THB	13.6
38	W	HB	10.7	P_THB	11.1
30	M	HB	15.2	P_THB	15.6
77	M	HB	14.9	P_THB	15.3
80	W	HB	12.7	P_THB	13.1
31	M	HB	15.2	P_THB	15.6
53	M	HB	12.9	P_THB	13.3
89	M	HB	16.1	P_THB	15.7
39	W	HB	13.2	P_THB	13.6
47	W	HB	13.9	P_THB	14.3
79	W	HB	9.9	P_THB	10.3
75	M	HB	13.2	P_THB	13.6
56	W	HB	12.4	P_THB	12.8
23	W	HB	13.7	P_THB	14.1
39	W	HB	11.7	P_THB	12.1
87	W	HB	8.4	P_THB	8.8
25	W	HB	10.9	P_THB	11.3
63	W	HB	13.1	P_THB	12.7
76	M	HB	12.7	P_THB	13.1
74	W	HB	10.9	P_THB	11.3
27	M	HB	15.8	P_THB	16.2
75	W	HB	14.9	P_THB	15.3
77	M	HB	14.4	P_THB	14.8
29	M	HB	15.7	P_THB	16.1
25	M	HB	14.9	P_THB	15.3
66	M	HB	8.4	P_THB	8.8
50	M	HB	15.7	P_THB	16.1
39	W	HB	12.4	P_THB	12.8
47	M	HB	14.9	P_THB	15.3
22	M	HB	11.4	P_THB	11.8
74	M	HB	14.4	P_THB	14.8
48	M	HB	14.9	P_THB	15.3
60	W	HB	13.7	P_THB	14.1
82	M	HB	10.9	P_THB	11.3
60	W	HB	13.9	P_THB	14.3
58	M	HB	15.1	P_THB	14.7
51	W	HB	14.2	P_THB	14.6
31	W	HB	9.2	P_THB	9.6
70	W	HB	10.4	P_THB	10.8
53	M	HB	12.8	P_THB	12.4
50	M	HB	13.9	P_THB	14.3
56	W	HB	11.2	P_THB	11.6
28	M	HB	15.2	P_THB	15.6
88	M	HB	11.4	P_THB	11.8
66	M	HB	13.6	P_THB	13.2
64	W	HB	12.9	P_THB	13.3
19	W	HB	13.2	P_THB	13.6
23	W	HB	12.4	P_THB	12.8

45	M	HB	13.9	P_THB	14.3
83	M	HB	11.4	P_THB	11.8
78	W	HB	11.7	P_THB	12.1
19	M	HB	15.8	P_THB	16.2
27	M	HB	14.9	P_THB	15.3
62	M	HB	14.2	P_THB	14.6
63	W	HB	13.7	P_THB	14.1
89	W	HB	9.9	P_THB	10.3
63	M	HB	15.8	P_THB	16.3
53	W	HB	15.8	P_THB	16.3
57	W	HB	15.8	P_THB	16.3
17	M	HB	15.8	P_THB	16.3
25	M	HB	15.8	P_THB	16.3
33	M	HB	15.8	P_THB	16.3
62	M	HB	8.4	P_THB	7.9
50	W	HB	7.9	P_THB	8.4
71	W	HB	10.9	P_THB	11.4
74	M	HB	16.4	P_THB	16.9
86	M	HB	13.1	P_THB	13.6
59	M	HB	14.7	P_THB	15.2
68	W	HB	13.9	P_THB	14.4
38	M	HB	16.0	P_THB	16.5
93	M	HB	13.6	P_THB	13.1
32	W	HB	14.4	P_THB	14.9
60	W	HB	12.8	P_THB	13.3
40	M	HB	13.9	P_THB	14.4
25	M	HB	14.0	P_THB	14.5
79	W	HB	15.3	P_THB	15.8
47	W	HB	11.0	P_THB	11.5
44	M	HB	8.5	P_THB	9.0
61	M	HB	13.3	P_THB	12.8
53	M	HB	14.3	P_THB	14.8
73	W	HB	12.6	P_THB	13.1
62	W	HB	11.8	P_THB	12.3
50	M	HB	12.9	P_THB	13.4
65	M	HB	14.8	P_THB	15.3
82	W	HB	10.8	P_THB	10.3
70	M	HB	12.6	P_THB	13.1
35	W	HB	15.3	P_THB	15.8
28	M	HB	14.3	P_THB	14.8
56	W	HB	12.8	P_THB	13.3
75	W	HB	14.1	P_THB	13.6
48	M	HB	12.4	P_THB	12.9
19	M	HB	15.5	P_THB	16.0
28	M	HB	16.1	P_THB	16.6
45	W	HB	13.8	P_THB	14.3
64	W	HB	14.7	P_THB	15.2
83	M	HB	13.1	P_THB	13.6
60	M	HB	14.3	P_THB	14.8
57	M	HB	11.7	P_THB	12.2
71	W	HB	11.5	P_THB	12.0
47	M	HB	14.6	P_THB	15.1
66	W	HB	13.1	P_THB	13.6
24	M	HB	15.1	P_THB	14.6
28	W	HB	11.5	P_THB	12.0
75	W	HB	9.8	P_THB	10.3

77	W	HB	13.1	P_THB	13.6
71	M	HB	10.8	P_THB	11.3
36	M	HB	14.8	P_THB	15.3
59	M	HB	14.4	P_THB	13.9
30	W	HB	13.2	P_THB	13.7
79	M	HB	14.1	P_THB	14.6
61	M	HB	11.6	P_THB	12.1
20	W	HB	14.4	P_THB	13.9
60	M	HB	14.2	P_THB	14.7
63	M	HB	13.7	P_THB	14.2
44	W	HB	14.2	P_THB	14.7
40	M	HB	15.3	P_THB	15.8
48	M	HB	14.6	P_THB	15.1
39	W	HB	10.9	P_THB	11.4
64	M	HB	10.5	P_THB	11.0
57	M	HB	16.1	P_THB	15.6
61	M	HB	14.6	P_THB	15.1
72	W	HB	14.0	P_THB	14.5
35	M	HB	15.3	P_THB	15.8
87	W	HB	14.3	P_THB	14.8
44	W	HB	13.0	P_THB	13.5
61	M	HB	14.8	P_THB	15.3
65	W	HB	12.0	P_THB	11.5
28	W	HB	12.8	P_THB	12.3
52	M	HB	16.8	P_THB	17.3
83	W	HB	13.1	P_THB	13.6
69	W	HB	14.5	P_THB	15.0
84	M	HB	13.1	P_THB	12.6
85	M	HB	11.0	P_THB	11.5
20	W	HB	11.9	P_THB	11.4
50	W	HB	13.5	P_THB	14.0
51	W	HB	13.2	P_THB	13.7
61	W	HB	12.3	P_THB	12.8
36	M	HB	15.9	P_THB	16.4
67	M	HB	13.1	P_THB	13.6
49	M	HB	16.5	P_THB	17.0
60	M	HB	14.5	P_THB	15.0
45	M	HB	11.8	P_THB	12.3
70	M	HB	13.6	P_THB	14.1
54	W	HB	14.3	P_THB	14.8
38	M	HB	15.2	P_THB	15.7
63	M	HB	14.4	P_THB	14.9
80	M	HB	12.3	P_THB	12.8
21	W	HB	14.4	P_THB	14.9
28	M	HB	15.4	P_THB	15.9
41	W	HB	13.1	P_THB	12.6
66	M	HB	13.7	P_THB	14.2
32	M	HB	14.0	P_THB	14.5
38	M	HB	15.4	P_THB	15.9
85	M	HB	15.2	P_THB	15.7
84	W	HB	9.1	P_THB	9.6
94	M	HB	11.4	P_THB	10.9
53	W	HB	12.2	P_THB	12.7
55	M	HB	11.6	P_THB	12.1
19	W	HB	14.4	P_THB	14.9
73	M	HB	15.1	P_THB	15.6

65	W	HB	9.3	P_THB	9.8
57	W	HB	9.7	P_THB	10.2
52	M	HB	14.2	P_THB	14.7
75	W	HB	11.1	P_THB	11.6
58	M	HB	6.1	P_THB	6.6
36	M	HB	13.8	P_THB	14.3
25	W	HB	12.6	P_THB	13.1
85	M	HB	15.2	P_THB	15.7
65	M	HB	13.9	P_THB	14.4
93	W	HB	13.5	P_THB	14.0
63	W	HB	14.1	P_THB	14.6
82	W	HB	12.4	P_THB	12.9
33	M	HB	14.0	P_THB	13.5
68	W	HB	13.4	P_THB	13.9
74	W	HB	13.9	P_THB	14.4
39	W	HB	12.6	P_THB	13.1
61	W	HB	13.9	P_THB	14.4
44	M	HB	14.0	P_THB	14.5
41	W	HB	14.1	P_THB	14.6
47	M	HB	14.0	P_THB	14.5
70	W	HB	12.3	P_THB	12.8
53	W	HB	13.9	P_THB	14.4
72	W	HB	12.0	P_THB	12.5
39	M	HB	12.3	P_THB	12.8
61	W	HB	13.2	P_THB	13.7
81	M	HB	14.2	P_THB	14.7
64	W	HB	13.7	P_THB	14.2
84	W	HB	10.5	P_THB	11.0
17	M	HB	16.0	P_THB	16.5
21	W	HB	14.8	P_THB	15.3
29	W	HB	12.7	P_THB	13.2
21	W	HB	12.9	P_THB	13.4
70	W	HB	13.4	P_THB	13.9
44	M	HB	14.7	P_THB	15.2
50	M	HB	10.9	P_THB	11.4
44	M	HB	15.6	P_THB	16.1
68	M	HB	16.0	P_THB	16.5
62	W	HB	12.9	P_THB	13.4
53	M	HB	14.3	P_THB	14.8
40	W	HB	11.8	P_THB	12.3
69	M	HB	15.0	P_THB	15.5
78	W	HB	13.3	P_THB	13.8
75	M	HB	14.6	P_THB	15.1
54	W	HB	14.5	P_THB	15.0
22	W	HB	14.2	P_THB	14.7
53	M	HB	13.6	P_THB	14.1
74	M	HB	16.6	P_THB	17.1
58	M	HB	13.4	P_THB	13.9
82	M	HB	10.3	P_THB	10.8
68	M	HB	13.1	P_THB	13.6
52	W	HB	11.8	P_THB	12.3
74	M	HB	12.2	P_THB	12.7
52	M	HB	13.5	P_THB	14.0
78	W	HB	16.5	P_THB	17.0
51	M	HB	13.2	P_THB	13.7
35	M	HB	8.7	P_THB	9.2

74	M	HB	14.9	P_THB	14.4
44	W	HB	11.6	P_THB	12.1
61	M	HB	15.4	P_THB	14.9
58	W	HB	8.6	P_THB	9.1
69	W	HB	14.0	P_THB	14.5
81	M	HB	13.1	P_THB	13.6
34	M	HB	12.4	P_THB	12.9
88	W	HB	11.7	P_THB	12.2
43	M	HB	13.1	P_THB	13.6
54	M	HB	15.5	P_THB	16.0
67	W	HB	13.9	P_THB	14.4
36	M	HB	13.1	P_THB	13.6
15	W	HB	14.5	P_THB	15.0
49	W	HB	12.1	P_THB	12.6
50	W	HB	14.1	P_THB	14.6
77	M	HB	11.2	P_THB	11.7
47	M	HB	14.8	P_THB	15.3
87	W	HB	11.6	P_THB	12.1
27	W	HB	13.1	P_THB	13.6
70	M	HB	14.5	P_THB	15.0
20	M	HB	18.0	P_THB	18.5
36	M	HB	16.3	P_THB	16.8
47	M	HB	14.9	P_THB	15.4
67	M	HB	14.9	P_THB	15.4
78	W	HB	14.2	P_THB	13.7
80	W	HB	11.1	P_THB	10.6
24	W	HB	12.3	P_THB	12.8
30	M	HB	15.1	P_THB	15.6
22	M	HB	16.2	P_THB	16.7
57	M	HB	15.0	P_THB	15.5
18	W	HB	13.5	P_THB	14.0
46	M	HB	15.5	P_THB	16.0
23	M	HB	13.8	P_THB	14.3
73	M	HB	14.3	P_THB	13.8
56	M	HB	15.5	P_THB	16.0
89	W	HB	12.0	P_THB	11.5
46	M	HB	16.1	P_THB	15.6
52	W	HB	9.6	P_THB	10.1
24	M	HB	14.8	P_THB	15.3
68	M	HB	15.2	P_THB	15.7
39	W	HB	13.5	P_THB	14.0
50	M	HB	16.3	P_THB	16.8
66	W	HB	12.5	P_THB	13.0
47	M	HB	14.8	P_THB	14.3
43	W	HB	13.7	P_THB	14.2
25	W	HB	14.8	P_THB	15.3
60	W	HB	12.1	P_THB	12.6
32	W	HB	12.3	P_THB	12.8
77	W	HB	13.1	P_THB	13.6
43	M	HB	15.5	P_THB	16.0
63	M	HB	13.4	P_THB	12.9
80	M	HB	11.8	P_THB	12.3
80	M	HB	15.2	P_THB	15.7
73	M	HB	10.6	P_THB	11.1
60	W	HB	10.9	P_THB	11.4
53	M	HB	11.2	P_THB	11.7

48	W	HB	12.6	P_THB	13.1
75	W	HB	13.1	P_THB	13.6
35	W	HB	13.7	P_THB	14.2
46	M	HB	14.8	P_THB	15.3
61	M	HB	13.4	P_THB	13.9
57	W	HB	12.3	P_THB	12.8
31	W	HB	13.9	P_THB	14.4
49	M	HB	16.0	P_THB	16.5
69	W	HB	15.9	P_THB	16.4
83	W	HB	10.2	P_THB	9.7
62	M	HB	14.0	P_THB	14.5
68	W	HB	9.5	P_THB	10.0
67	M	HB	14.7	P_THB	15.2
21	W	HB	12.9	P_THB	13.4
85	M	HB	12.0	P_THB	12.5
58	M	HB	11.5	P_THB	11.0
60	W	HB	9.2	P_THB	9.7
76	M	HB	13.7	P_THB	14.2
33	W	HB	12.1	P_THB	12.6
72	M	HB	7.5	P_THB	8.0
67	M	HB	14.3	P_THB	14.8
48	W	HB	12.0	P_THB	12.5
31	M	HB	14.9	P_THB	15.4
52	W	HB	14.9	P_THB	15.4
41	M	HB	12.8	P_THB	13.3
14	M	HB	15.3	P_THB	15.8
64	M	HB	15.5	P_THB	16.0
47	M	HB	9.3	P_THB	9.8
75	W	HB	13.0	P_THB	13.5
69	W	HB	9.7	P_THB	10.2
85	W	HB	12.3	P_THB	12.8
55	M	HB	8.3	P_THB	8.8
24	M	HB	15.0	P_THB	15.5
93	W	HB	13.0	P_THB	12.5
48	M	HB	16.3	P_THB	16.8
56	W	HB	12.9	P_THB	13.4
21	W	HB	13.1	P_THB	13.6
90	M	HB	10.9	P_THB	11.4
32	W	HB	12.4	P_THB	12.9
48	M	HB	14.7	P_THB	15.2
20	W	HB	13.3	P_THB	13.8
68	M	HB	12.4	P_THB	12.9
40	W	HB	13.8	P_THB	14.3
72	M	HB	15.1	P_THB	15.6
70	M	HB	11.7	P_THB	12.2
92	W	HB	8.4	P_THB	8.9
82	W	HB	13.3	P_THB	13.8
78	M	HB	10.3	P_THB	10.8
24	W	HB	13.8	P_THB	14.3
64	M	HB	11.8	P_THB	12.3
74	M	HB	15.0	P_THB	15.5
44	M	HB	14.5	P_THB	15.0
28	W	HB	13.4	P_THB	13.9
67	M	HB	8.1	P_THB	8.6
37	M	HB	15.4	P_THB	15.9
44	M	HB	13.9	P_THB	14.4

57	W	HB	12.2	P_THB	12.7
82	W	HB	12.0	P_THB	12.5
73	M	HB	12.0	P_THB	12.5
70	W	HB	10.6	P_THB	11.1
49	M	HB	13.2	P_THB	13.7
57	M	HB	9.1	P_THB	9.6
56	M	HB	12.4	P_THB	12.9
48	W	HB	10.0	P_THB	10.5
78	M	HB	11.4	P_THB	11.9
78	M	HB	10.8	P_THB	11.3
32	M	HB	16.1	P_THB	16.6
88	W	HB	12.6	P_THB	13.1
50	W	HB	14.2	P_THB	14.7
79	W	HB	13.7	P_THB	14.2
87	M	HB	13.8	P_THB	13.3
60	W	HB	14.9	P_THB	15.4
61	M	HB	15.3	P_THB	15.8
64	M	HB	13.1	P_THB	13.6
84	W	HB	11.4	P_THB	11.9
56	M	HB	17.4	P_THB	17.9
19	W	HB	12.3	P_THB	12.8
24	W	HB	12.9	P_THB	13.4
57	M	HB	11.0	P_THB	11.5
67	M	HB	13.4	P_THB	12.9
22	M	HB	14.8	P_THB	15.3
36	W	HB	13.4	P_THB	13.9
74	W	HB	9.3	P_THB	9.8
74	M	HB	13.3	P_THB	13.8
90	W	HB	11.8	P_THB	12.3
62	M	HB	15.6	P_THB	16.1
76	M	HB	11.8	P_THB	12.3
36	W	HB	13.2	P_THB	13.7
52	M	HB	12.7	P_THB	13.2
60	W	HB	13.7	P_THB	14.2
55	W	HB	12.8	P_THB	13.3
80	W	HB	14.5	P_THB	15.0
92	W	HB	13.7	P_THB	14.2
88	W	HB	12.5	P_THB	13.0
39	M	HB	14.6	P_THB	15.1
80	W	HB	13.0	P_THB	13.5
43	M	HB	16.0	P_THB	16.5
40	W	HB	12.8	P_THB	13.3
93	W	HB	8.1	P_THB	8.6
73	M	HB	15.2	P_THB	15.7
42	M	HB	12.7	P_THB	12.2
33	M	HB	14.9	P_THB	15.4
86	W	HB	12.5	P_THB	13.0
52	M	HB	9.8	P_THB	10.3
53	W	HB	13.4	P_THB	13.9
22	M	HB	14.7	P_THB	15.2
60	M	HB	14.9	P_THB	15.4
21	W	HB	12.9	P_THB	13.4
19	W	HB	13.5	P_THB	14.0
42	W	HB	13.6	P_THB	14.1
93	W	HB	9.2	P_THB	9.7
63	M	HB	10.8	P_THB	11.3

84	M	HB	11.2	P_THB	11.7
74	W	HB	7.6	P_THB	8.1
60	W	HB	16.2	P_THB	15.7
25	M	HB	15.7	P_THB	16.2
53	M	HB	15.7	P_THB	16.2
31	M	HB	15.7	P_THB	16.2
74	M	HB	16.7	P_THB	17.3
58	M	HB	16.2	P_THB	16.8
36	M	HB	16.7	P_THB	17.3
70	M	HB	16.7	P_THB	17.3
53	M	HB	16.7	P_THB	17.3
37	M	HB	16.2	P_THB	16.8
28	M	HB	16.2	P_THB	16.8
57	M	HB	12.8	P_THB	13.4
55	M	HB	11.1	P_THB	11.7
34	W	HB	13.6	P_THB	14.2
70	M	HB	12.1	P_THB	12.7
57	W	HB	11.3	P_THB	11.9
85	W	HB	12.6	P_THB	13.2
89	W	HB	15.3	P_THB	15.9
57	M	HB	15.1	P_THB	15.7
91	W	HB	13.3	P_THB	13.9
58	M	HB	12.6	P_THB	13.2
68	M	HB	10.3	P_THB	10.9
26	M	HB	15.7	P_THB	16.3
37	W	HB	12.1	P_THB	12.7
26	W	HB	13.8	P_THB	14.4
16	M	HB	13.8	P_THB	14.4
36	W	HB	13.1	P_THB	13.7
64	M	HB	14.7	P_THB	14.1
75	M	HB	14.8	P_THB	15.4
72	M	HB	10.6	P_THB	11.2
70	M	HB	15.7	P_THB	16.3
35	M	HB	14.3	P_THB	14.9
30	W	HB	13.8	P_THB	14.4
60	M	HB	14.3	P_THB	14.9
36	M	HB	14.8	P_THB	15.4
58	M	HB	14.8	P_THB	15.4
87	M	HB	12.8	P_THB	13.4
75	M	HB	12.3	P_THB	12.9
56	M	HB	16.3	P_THB	15.7
24	W	HB	15.3	P_THB	15.9
75	M	HB	12.8	P_THB	13.4
78	M	HB	14.3	P_THB	14.9
47	M	HB	12.8	P_THB	13.4
39	M	HB	13.6	P_THB	14.2
80	W	HB	12.6	P_THB	13.2
73	M	HB	12.8	P_THB	13.4
61	M	HB	12.3	P_THB	12.9
42	M	HB	13.6	P_THB	14.2
50	M	HB	15.8	P_THB	16.4
75	M	HB	14.6	P_THB	15.2
39	M	HB	13.8	P_THB	14.4
74	W	HB	13.4	P_THB	12.8
23	W	HB	9.6	P_THB	10.2
7	M	HB	12.3	P_THB	12.9

99	W	HB	11.3	P_THB	11.9
91	W	HB	10.8	P_THB	11.4
60	W	HB	8.6	P_THB	9.2
79	M	HB	14.1	P_THB	14.7
38	W	HB	14.8	P_THB	15.4
50	M	HB	15.1	P_THB	15.7
75	W	HB	8.8	P_THB	9.4
49	W	HB	14.1	P_THB	14.7
84	M	HB	13.6	P_THB	14.2
72	M	HB	11.1	P_THB	11.7
25	W	HB	12.6	P_THB	13.2
27	W	HB	10.8	P_THB	11.4
75	W	HB	13.8	P_THB	14.4
30	M	HB	15.7	P_THB	15.1
69	M	HB	14.3	P_THB	14.9
78	W	HB	12.1	P_THB	12.7
49	W	HB	13.6	P_THB	14.2
50	W	HB	15.4	P_THB	14.8
78	W	HB	12.1	P_THB	12.7
63	M	HB	14.3	P_THB	14.9
19	W	HB	11.3	P_THB	11.9
21	M	HB	14.6	P_THB	15.2
22	W	HB	14.1	P_THB	14.7
49	M	HB	15.1	P_THB	15.7
77	M	HB	13.1	P_THB	13.7
57	W	HB	13.6	P_THB	14.2
74	W	HB	15.7	P_THB	16.3
28	M	HB	15.8	P_THB	16.4
51	M	HB	12.6	P_THB	13.2
41	M	HB	14.8	P_THB	15.4
42	M	HB	14.1	P_THB	14.7
72	W	HB	11.3	P_THB	11.9
83	W	HB	9.7	P_THB	9.1
29	M	HB	14.8	P_THB	15.4
59	M	HB	13.6	P_THB	14.2
57	W	HB	11.8	P_THB	12.4
84	M	HB	13.6	P_THB	14.2
83	W	HB	14.8	P_THB	15.4
77	M	HB	13.7	P_THB	13.1
20	W	HB	12.3	P_THB	12.9
52	W	HB	12.1	P_THB	12.7
50	W	HB	12.6	P_THB	13.2
85	M	HB	14.6	P_THB	15.2
50	W	HB	12.8	P_THB	13.4
53	W	HB	12.3	P_THB	12.9
16	M	HB	14.8	P_THB	15.4
28	W	HB	13.2	P_THB	12.6
43	M	HB	12.8	P_THB	13.4
80	M	HB	11.6	P_THB	12.2
55	M	HB	14.8	P_THB	15.4
55	M	HB	13.3	P_THB	13.9
62	M	HB	12.8	P_THB	13.4
85	W	HB	10.6	P_THB	11.2
48	M	HB	12.8	P_THB	13.4
28	W	HB	12.1	P_THB	12.7
42	M	HB	7.8	P_THB	8.4

82	M	HB	9.6	P_THB	10.2
90	W	HB	10.8	P_THB	11.4
60	W	HB	11.1	P_THB	11.7
39	M	HB	14.7	P_THB	14.1
58	M	HB	16.4	P_THB	15.8
86	W	HB	12.1	P_THB	12.7
44	W	HB	11.8	P_THB	12.4
47	M	HB	13.8	P_THB	14.4
65	W	HB	10.3	P_THB	10.9
29	W	HB	14.1	P_THB	14.7
66	M	HB	15.1	P_THB	15.7
77	W	HB	13.3	P_THB	13.9
48	M	HB	13.6	P_THB	14.2
46	W	HB	14.3	P_THB	14.9
53	M	HB	14.1	P_THB	14.7
53	W	HB	12.6	P_THB	13.2
82	M	HB	9.1	P_THB	9.7
53	M	HB	11.6	P_THB	12.2
49	M	HB	15.7	P_THB	16.3
60	M	HB	10.8	P_THB	11.4
24	W	HB	12.8	P_THB	13.4
40	M	HB	14.8	P_THB	15.4
86	M	HB	11.6	P_THB	12.2
27	M	HB	9.3	P_THB	9.9
44	W	HB	13.6	P_THB	14.2
31	M	HB	15.7	P_THB	16.3
74	M	HB	13.3	P_THB	13.9
84	M	HB	13.6	P_THB	14.2
56	M	HB	15.3	P_THB	15.9
76	W	HB	11.3	P_THB	11.9
57	M	HB	10.1	P_THB	10.7
89	M	HB	10.6	P_THB	11.2
39	W	HB	14.3	P_THB	14.9
73	M	HB	14.1	P_THB	14.7
57	W	HB	13.1	P_THB	13.7
25	M	HB	12.3	P_THB	12.9
79	W	HB	13.3	P_THB	13.9
60	W	HB	12.6	P_THB	13.2
65	M	HB	10.8	P_THB	11.4
42	W	HB	12.3	P_THB	12.9
25	W	HB	14.8	P_THB	15.4
47	M	HB	10.1	P_THB	10.7
74	M	HB	9.8	P_THB	10.4
71	M	HB	10.3	P_THB	10.9
59	W	HB	15.1	P_THB	15.7
40	M	HB	8.2	P_THB	7.6
31	M	HB	13.9	P_THB	14.5
68	M	HB	10.7	P_THB	11.3
62	M	HB	16.9	P_THB	17.5
61	W	HB	13.5	P_THB	14.1
58	M	HB	15.5	P_THB	16.1
68	W	HB	14.0	P_THB	14.6
17	W	HB	8.9	P_THB	9.5
75	M	HB	10.4	P_THB	11.0
45	M	HB	13.7	P_THB	14.3
24	M	HB	16.0	P_THB	16.6

56	M	HB	16.4	P_THB	17.0
36	W	HB	11.9	P_THB	12.5
54	W	HB	13.4	P_THB	14.0
85	M	HB	10.2	P_THB	10.8
77	W	HB	13.0	P_THB	13.6
68	M	HB	12.5	P_THB	13.1
41	W	HB	15.0	P_THB	15.6
82	W	HB	12.7	P_THB	13.3
64	W	HB	12.5	P_THB	13.1
81	M	HB	11.7	P_THB	12.3
42	M	HB	14.4	P_THB	15.0
25	W	HB	13.7	P_THB	14.3
69	M	HB	10.7	P_THB	11.3
89	W	HB	11.5	P_THB	12.1
83	W	HB	10.9	P_THB	11.5
92	M	HB	9.2	P_THB	9.8
76	W	HB	9.4	P_THB	10.0
46	M	HB	16.0	P_THB	16.6
26	M	HB	14.2	P_THB	14.8
52	M	HB	13.9	P_THB	14.5
81	M	HB	12.4	P_THB	13.0
43	W	HB	14.2	P_THB	14.8
32	W	HB	12.2	P_THB	12.8
24	W	HB	12.7	P_THB	13.3
55	W	HB	11.9	P_THB	12.5
77	W	HB	12.7	P_THB	13.3
56	M	HB	13.9	P_THB	14.5
72	W	HB	11.2	P_THB	11.8
91	M	HB	15.4	P_THB	16.0
32	M	HB	16.0	P_THB	16.6
34	M	HB	14.6	P_THB	14.0
32	W	HB	12.4	P_THB	13.0
21	M	HB	16.6	P_THB	17.2
64	M	HB	13.7	P_THB	14.3
26	W	HB	13.9	P_THB	14.5
45	M	HB	14.9	P_THB	15.5
71	M	HB	9.9	P_THB	10.5
53	M	HB	17.3	P_THB	17.9
69	M	HB	12.9	P_THB	13.5
79	M	HB	8.7	P_THB	9.3
35	M	HB	14.7	P_THB	15.3
80	M	HB	15.2	P_THB	15.8
60	M	HB	13.7	P_THB	14.3
41	W	HB	15.6	P_THB	16.2
22	W	HB	13.5	P_THB	14.1
36	W	HB	13.5	P_THB	14.1
55	M	HB	14.7	P_THB	15.3
54	M	HB	14.7	P_THB	15.3
92	W	HB	15.0	P_THB	15.6
87	M	HB	12.9	P_THB	13.5
88	M	HB	12.5	P_THB	13.1
39	M	HB	14.4	P_THB	15.0
38	M	HB	15.4	P_THB	16.0
45	M	HB	14.5	P_THB	15.1
45	W	HB	8.2	P_THB	8.8
29	W	HB	11.9	P_THB	12.5

63	M	HB	14.5	P_THB	15.1
85	W	HB	14.9	P_THB	15.5
21	W	HB	13.4	P_THB	14.0
45	M	HB	14.7	P_THB	15.3
69	M	HB	12.9	P_THB	13.5
77	M	HB	14.0	P_THB	14.6
33	M	HB	15.2	P_THB	15.8
43	M	HB	15.2	P_THB	15.8
93	M	HB	13.0	P_THB	13.6
79	W	HB	14.9	P_THB	15.5
83	W	HB	10.5	P_THB	11.1
55	M	HB	15.2	P_THB	15.8
41	M	HB	16.1	P_THB	16.7
25	M	HB	14.4	P_THB	15.0
21	M	HB	13.4	P_THB	14.0
47	W	HB	10.4	P_THB	11.0
52	M	HB	15.8	P_THB	15.2
44	W	HB	14.7	P_THB	15.3
53	W	HB	14.2	P_THB	14.8
46	W	HB	15.0	P_THB	15.6
84	W	HB	11.0	P_THB	11.6
77	W	HB	9.4	P_THB	10.0
49	W	HB	13.5	P_THB	14.1
28	W	HB	13.0	P_THB	13.6
66	W	HB	9.2	P_THB	9.8
44	M	HB	14.5	P_THB	15.1
67	W	HB	11.9	P_THB	12.5
83	W	HB	11.7	P_THB	12.3
15	M	HB	14.5	P_THB	15.1
59	M	HB	13.5	P_THB	14.1
58	M	HB	11.5	P_THB	12.1
80	M	HB	14.4	P_THB	15.0
76	M	HB	16.6	P_THB	17.2
43	M	HB	11.4	P_THB	12.0
50	W	HB	14.2	P_THB	14.8
91	M	HB	9.7	P_THB	10.3
74	M	HB	14.2	P_THB	14.8
86	M	HB	14.5	P_THB	15.1
74	M	HB	12.2	P_THB	12.8
53	M	HB	12.5	P_THB	13.1
75	W	HB	13.4	P_THB	14.0
115	M	HB	12.4	P_THB	13.0
36	W	HB	12.0	P_THB	12.6
69	W	HB	9.9	P_THB	10.5
73	W	HB	14.4	P_THB	15.0
62	M	HB	9.0	P_THB	9.6
74	M	HB	8.9	P_THB	9.5
23	M	HB	14.2	P_THB	14.8
52	W	HB	13.0	P_THB	13.6
87	M	HB	13.5	P_THB	14.1
80	W	HB	13.0	P_THB	13.6
52	W	HB	13.9	P_THB	14.5
59	W	HB	16.8	P_THB	17.4
57	M	HB	15.6	P_THB	15.0
46	W	HB	16.3	P_THB	16.9
53	M	HB	13.9	P_THB	14.5

24	W	HB	13.4	P_THB	14.0
79	M	HB	11.7	P_THB	12.3
29	W	HB	14.5	P_THB	15.1
82	W	HB	12.4	P_THB	13.0
46	M	HB	15.9	P_THB	16.5
27	M	HB	14.2	P_THB	14.8
28	W	HB	12.7	P_THB	13.3
40	W	HB	9.5	P_THB	10.1
51	W	HB	13.4	P_THB	14.0
84	M	HB	11.4	P_THB	12.0
89	W	HB	12.4	P_THB	13.0
33	W	HB	15.9	P_THB	16.5
92	M	HB	12.4	P_THB	13.0
86	W	HB	15.9	P_THB	16.5
54	W	HB	13.7	P_THB	14.3
50	M	HB	14.9	P_THB	15.5
64	M	HB	9.0	P_THB	9.6
35	W	HB	13.2	P_THB	13.8
83	M	HB	12.2	P_THB	12.8
37	W	HB	12.0	P_THB	12.6
17	W	HB	13.7	P_THB	14.3
85	W	HB	13.4	P_THB	14.0
79	W	HB	12.2	P_THB	12.8
38	W	HB	13.7	P_THB	14.3
11	W	HB	13.0	P_THB	13.6
78	M	HB	16.6	P_THB	17.2
37	M	HB	13.4	P_THB	14.0
89	W	HB	13.0	P_THB	13.6
33	W	HB	14.2	P_THB	14.8
57	W	HB	14.8	P_THB	14.2
65	M	HB	10.7	P_THB	11.3
73	M	HB	13.5	P_THB	14.1
78	W	HB	13.0	P_THB	13.6
66	W	HB	14.7	P_THB	15.3
51	M	HB	15.5	P_THB	16.1
55	M	HB	14.9	P_THB	15.5
54	M	HB	14.4	P_THB	15.0
72	M	HB	12.4	P_THB	13.0
31	W	HB	13.4	P_THB	14.0
17	W	HB	13.5	P_THB	14.1
32	M	HB	16.8	P_THB	17.4
37	M	HB	15.3	P_THB	14.7
20	M	HB	15.6	P_THB	15.0
75	W	HB	10.9	P_THB	11.5
27	M	HB	15.4	P_THB	16.0
28	M	HB	14.0	P_THB	13.4
71	W	HB	13.9	P_THB	14.5
42	W	HB	13.5	P_THB	14.1
90	W	HB	11.7	P_THB	12.3
37	M	HB	15.9	P_THB	16.5
45	M	HB	10.7	P_THB	11.3
72	W	HB	12.2	P_THB	12.8
81	M	HB	11.4	P_THB	12.0
24	W	HB	13.5	P_THB	14.1
63	M	HB	15.2	P_THB	15.8
47	W	HB	14.0	P_THB	14.6

34	M	HB	14.9	P_THB	15.5
16	M	HB	13.9	P_THB	14.5
46	W	HB	14.0	P_THB	14.6
48	W	HB	15.9	P_THB	16.5
31	W	HB	12.7	P_THB	13.3
72	M	HB	10.4	P_THB	11.0
33	M	HB	14.7	P_THB	15.3
52	M	HB	14.4	P_THB	15.0
35	W	HB	12.4	P_THB	13.0
25	W	HB	12.0	P_THB	12.6
58	W	HB	14.5	P_THB	15.1
71	W	HB	12.0	P_THB	12.6
65	M	HB	14.4	P_THB	15.0
79	M	HB	14.9	P_THB	15.5
52	W	HB	13.2	P_THB	13.8
49	M	HB	15.2	P_THB	15.8
52	W	HB	12.9	P_THB	13.5
91	W	HB	12.7	P_THB	13.3
68	M	HB	14.7	P_THB	15.3
52	M	HB	15.0	P_THB	15.6
24	M	HB	16.6	P_THB	17.2
43	M	HB	15.0	P_THB	15.6
37	M	HB	14.4	P_THB	15.0
23	W	HB	11.0	P_THB	11.6
42	M	HB	8.0	P_THB	8.6
84	W	HB	13.7	P_THB	14.3
34	M	HB	14.0	P_THB	13.4
27	M	HB	15.0	P_THB	15.6
35	W	HB	12.7	P_THB	13.3
56	M	HB	12.9	P_THB	13.5
82	M	HB	10.9	P_THB	11.5
71	W	HB	9.5	P_THB	10.1
35	W	HB	11.2	P_THB	11.8
72	W	HB	12.4	P_THB	13.0
36	M	HB	15.2	P_THB	15.8
73	M	HB	10.5	P_THB	11.1
37	W	HB	13.9	P_THB	14.5
65	M	HB	16.0	P_THB	16.6
72	W	HB	13.9	P_THB	14.5
54	M	HB	14.4	P_THB	15.0
59	M	HB	16.0	P_THB	16.6
81	M	HB	10.2	P_THB	10.8
77	W	HB	7.7	P_THB	8.3
22	W	HB	15.0	P_THB	15.6
64	W	HB	12.0	P_THB	12.6
48	M	HB	15.6	P_THB	16.2
65	M	HB	15.2	P_THB	15.8
77	W	HB	7.2	P_THB	7.8
61	W	HB	14.5	P_THB	15.1
41	M	HB	16.1	P_THB	16.7
86	M	HB	13.7	P_THB	14.3
45	M	HB	17.8	P_THB	18.4
85	W	HB	13.2	P_THB	13.8
61	M	HB	16.0	P_THB	16.6
63	M	HB	15.0	P_THB	14.4
62	M	HB	12.5	P_THB	13.1

76	W	HB	13.4	P_THB	14.0
61	M	HB	14.7	P_THB	15.3
33	M	HB	15.0	P_THB	15.6
46	M	HB	15.9	P_THB	16.5
67	M	HB	13.2	P_THB	13.8
46	W	HB	12.0	P_THB	12.6
89	W	HB	12.0	P_THB	12.6
66	W	HB	10.7	P_THB	11.3
79	W	HB	14.5	P_THB	15.1
45	M	HB	12.9	P_THB	13.5
55	W	HB	11.5	P_THB	12.1
84	M	HB	11.4	P_THB	12.0
51	W	HB	13.7	P_THB	14.3
73	M	HB	14.9	P_THB	15.5
47	W	HB	12.9	P_THB	13.5
27	W	HB	13.5	P_THB	14.1
47	M	HB	15.6	P_THB	16.2
54	W	HB	13.5	P_THB	14.1
80	M	HB	13.4	P_THB	14.0
72	W	HB	15.9	P_THB	16.5
22	W	HB	12.7	P_THB	13.3
45	W	HB	13.7	P_THB	14.3
78	M	HB	11.0	P_THB	11.6
77	M	HB	8.9	P_THB	9.5
82	W	HB	9.9	P_THB	10.5
73	M	HB	16.6	P_THB	17.3
66	W	HB	16.6	P_THB	17.3
54	M	HB	15.6	P_THB	16.3
48	M	HB	16.1	P_THB	16.8
69	M	HB	15.6	P_THB	16.3
17	M	HB	16.2	P_THB	16.9
33	M	HB	17.1	P_THB	17.8
47	M	HB	15.6	P_THB	16.3
46	M	HB	15.6	P_THB	16.3
56	M	HB	15.6	P_THB	16.3
23	M	HB	15.6	P_THB	16.3
20	M	HB	15.2	P_THB	15.9
32	M	HB	15.3	P_THB	16.0
77	W	HB	14.3	P_THB	15.0
79	M	HB	14.2	P_THB	14.9
71	W	HB	10.5	P_THB	11.2
75	W	HB	12.6	P_THB	13.3
52	W	HB	13.5	P_THB	14.2
21	W	HB	13.1	P_THB	13.8
46	M	HB	10.5	P_THB	11.2
24	M	HB	13.3	P_THB	14.0
58	W	HB	8.8	P_THB	9.5
48	M	HB	13.2	P_THB	13.9
59	M	HB	15.0	P_THB	15.7
21	W	HB	12.3	P_THB	13.0
30	W	HB	14.0	P_THB	14.7
23	M	HB	15.1	P_THB	15.8
17	W	HB	13.2	P_THB	13.9
69	W	HB	14.3	P_THB	13.6
75	W	HB	13.0	P_THB	13.7
92	M	HB	8.6	P_THB	9.3

25	M	HB	14.5	P_THB	15.2
55	M	HB	14.2	P_THB	14.9
22	W	HB	12.5	P_THB	13.2
20	W	HB	13.3	P_THB	14.0
32	M	HB	15.8	P_THB	16.5
92	W	HB	11.7	P_THB	12.4
54	W	HB	12.2	P_THB	12.9
82	M	HB	13.0	P_THB	12.3
44	W	HB	13.6	P_THB	14.3
59	W	HB	14.3	P_THB	15.0
54	M	HB	14.5	P_THB	15.2
87	M	HB	11.5	P_THB	12.2
66	W	HB	9.6	P_THB	10.3
82	W	HB	13.6	P_THB	14.3
85	W	HB	8.2	P_THB	8.9
81	W	HB	13.0	P_THB	13.7
82	W	HB	9.7	P_THB	10.4
38	W	HB	13.0	P_THB	13.7
63	M	HB	13.6	P_THB	14.3
66	W	HB	8.5	P_THB	9.2
39	M	HB	14.0	P_THB	14.7
44	M	HB	10.5	P_THB	11.2
76	W	HB	11.7	P_THB	12.4
84	W	HB	12.3	P_THB	13.0
79	M	HB	11.7	P_THB	12.4
75	W	HB	13.8	P_THB	14.5
58	M	HB	14.2	P_THB	14.9
78	W	HB	12.6	P_THB	13.3
83	W	HB	11.5	P_THB	12.2
51	M	HB	13.8	P_THB	14.5
26	W	HB	13.2	P_THB	13.9
36	M	HB	13.8	P_THB	14.5
76	M	HB	12.1	P_THB	12.8
50	M	HB	14.5	P_THB	15.2
54	M	HB	14.8	P_THB	15.5
17	W	HB	12.2	P_THB	12.9
66	M	HB	11.7	P_THB	12.4
89	W	HB	13.2	P_THB	13.9
34	W	HB	13.6	P_THB	14.3
69	W	HB	8.0	P_THB	8.7
75	W	HB	11.8	P_THB	12.5
24	M	HB	15.8	P_THB	16.5
43	M	HB	13.5	P_THB	14.2
71	M	HB	8.8	P_THB	9.5
59	W	HB	11.7	P_THB	12.4
52	W	HB	13.1	P_THB	13.8
77	M	HB	9.5	P_THB	8.8
79	W	HB	12.1	P_THB	12.8
83	W	HB	11.1	P_THB	11.8
36	M	HB	13.8	P_THB	14.5
61	W	HB	13.6	P_THB	14.3
84	W	HB	12.0	P_THB	12.7
65	M	HB	8.0	P_THB	8.7
82	W	HB	11.2	P_THB	11.9
53	W	HB	12.1	P_THB	12.8
38	W	HB	12.2	P_THB	12.9

36	W	HB	10.7	P_THB	11.4
67	W	HB	14.2	P_THB	14.9
67	W	HB	10.5	P_THB	11.2
39	W	HB	12.5	P_THB	13.2
91	M	HB	9.3	P_THB	10.0
69	W	HB	10.8	P_THB	11.5
83	M	HB	12.2	P_THB	12.9
68	M	HB	15.1	P_THB	15.8
62	W	HB	14.0	P_THB	14.7
32	M	HB	15.0	P_THB	15.7
64	W	HB	12.6	P_THB	13.3
48	M	HB	11.0	P_THB	11.7
60	W	HB	14.0	P_THB	14.7
23	M	HB	14.7	P_THB	15.4
78	W	HB	13.6	P_THB	14.3
56	W	HB	14.0	P_THB	14.7
36	W	HB	13.1	P_THB	13.8
54	W	HB	14.7	P_THB	15.4
77	W	HB	11.7	P_THB	12.4
66	M	HB	11.6	P_THB	12.3
57	W	HB	12.1	P_THB	12.8
21	W	HB	13.1	P_THB	13.8
80	M	HB	10.4	P_THB	9.7
74	M	HB	15.1	P_THB	15.8
31	W	HB	12.1	P_THB	12.8
19	W	HB	13.6	P_THB	14.3
75	M	HB	10.5	P_THB	11.2
62	W	HB	8.0	P_THB	8.7
63	W	HB	13.1	P_THB	13.8
14	W	HB	10.7	P_THB	11.4
71	M	HB	11.2	P_THB	11.9
24	W	HB	13.7	P_THB	14.4
66	M	HB	10.8	P_THB	10.1
64	M	HB	10.7	P_THB	11.4
51	W	HB	13.5	P_THB	14.2
59	M	HB	14.4	P_THB	13.7
79	M	HB	15.0	P_THB	15.7
38	W	HB	10.2	P_THB	10.9
35	W	HB	11.8	P_THB	12.5
76	M	HB	15.3	P_THB	16.0
81	M	HB	11.8	P_THB	12.5
49	M	HB	14.5	P_THB	15.2
78	W	HB	11.5	P_THB	12.2
75	M	HB	11.3	P_THB	12.0
76	M	HB	15.0	P_THB	15.7
77	W	HB	9.8	P_THB	10.5
87	M	HB	12.3	P_THB	13.0
32	M	HB	13.3	P_THB	14.0
59	W	HB	13.3	P_THB	14.0
71	W	HB	10.3	P_THB	11.0
75	M	HB	11.5	P_THB	12.2
41	M	HB	14.8	P_THB	15.5
53	M	HB	15.7	P_THB	16.4
114	M	HB	14.8	P_THB	15.5
52	M	HB	15.8	P_THB	16.5
29	W	HB	13.5	P_THB	14.2

83	M	HB	14.2	P_THB	13.5
76	M	HB	13.7	P_THB	14.4
90	W	HB	14.1	P_THB	14.8
75	W	HB	14.3	P_THB	15.0
18	M	HB	14.6	P_THB	15.3
43	M	HB	12.7	P_THB	13.4
28	W	HB	12.5	P_THB	13.2
62	M	HB	15.1	P_THB	15.8
29	M	HB	14.0	P_THB	14.7
86	W	HB	12.8	P_THB	13.5
57	W	HB	12.2	P_THB	12.9
33	W	HB	13.5	P_THB	14.2
77	W	HB	10.8	P_THB	11.5
29	W	HB	12.2	P_THB	12.9
73	W	HB	13.0	P_THB	13.7
33	W	HB	14.3	P_THB	15.0
21	W	HB	12.5	P_THB	11.8
83	M	HB	12.3	P_THB	13.0
78	M	HB	14.6	P_THB	15.3
84	W	HB	13.7	P_THB	14.4
48	M	HB	10.2	P_THB	10.9
27	M	HB	13.6	P_THB	14.3
57	W	HB	13.3	P_THB	14.0
71	W	HB	9.7	P_THB	10.4
63	M	HB	11.1	P_THB	11.8
80	M	HB	14.3	P_THB	15.0
52	M	HB	12.6	P_THB	13.3
64	W	HB	14.2	P_THB	14.9
27	M	HB	15.2	P_THB	15.9
85	W	HB	10.2	P_THB	10.9
67	M	HB	14.1	P_THB	14.8
79	W	HB	9.3	P_THB	10.0
68	W	HB	12.6	P_THB	13.3
40	M	HB	14.5	P_THB	15.2
77	M	HB	13.7	P_THB	14.4
82	M	HB	14.5	P_THB	15.2
49	W	HB	14.0	P_THB	14.7
70	M	HB	15.2	P_THB	15.9
42	W	HB	11.7	P_THB	12.4
89	W	HB	12.5	P_THB	13.2
34	M	HB	15.8	P_THB	16.5
36	W	HB	10.8	P_THB	11.5
22	W	HB	14.3	P_THB	15.0
24	W	HB	8.8	P_THB	9.5
70	M	HB	14.5	P_THB	15.2
21	M	HB	12.8	P_THB	13.5
31	M	HB	15.7	P_THB	16.4
76	M	HB	11.2	P_THB	11.9
52	W	HB	5.3	P_THB	6.0
23	W	HB	12.8	P_THB	13.5
36	W	HB	14.6	P_THB	15.3
84	W	HB	11.3	P_THB	12.0
40	M	HB	15.2	P_THB	15.9
71	M	HB	13.8	P_THB	14.5
41	W	HB	11.2	P_THB	11.9
72	W	HB	13.2	P_THB	13.9

42	W	HB	10.5	P_THB	11.2
63	M	HB	12.5	P_THB	13.2
17	M	HB	14.6	P_THB	15.3
84	W	HB	15.2	P_THB	15.9
86	W	HB	12.7	P_THB	13.4
49	M	HB	13.0	P_THB	13.7
85	M	HB	10.6	P_THB	11.3
25	W	HB	13.2	P_THB	13.9
75	W	HB	12.5	P_THB	13.2
34	W	HB	12.6	P_THB	13.3
24	W	HB	13.7	P_THB	14.4
72	M	HB	13.8	P_THB	14.5
77	W	HB	10.6	P_THB	11.3
36	M	HB	11.8	P_THB	12.5
89	W	HB	12.3	P_THB	13.0
75	M	HB	12.1	P_THB	12.8
57	W	HB	14.6	P_THB	15.3
37	M	HB	13.6	P_THB	14.3
33	W	HB	14.0	P_THB	14.7
50	M	HB	15.0	P_THB	15.7
45	W	HB	12.2	P_THB	12.9
62	W	HB	9.1	P_THB	9.8
43	W	HB	10.2	P_THB	10.9
37	M	HB	14.1	P_THB	14.8
58	M	HB	13.0	P_THB	13.7
86	W	HB	11.6	P_THB	12.3
22	W	HB	10.3	P_THB	9.6
79	W	HB	8.2	P_THB	8.9
73	M	HB	13.0	P_THB	13.7
41	M	HB	13.6	P_THB	14.3
81	M	HB	12.1	P_THB	12.8
39	W	HB	12.2	P_THB	12.9
73	M	HB	15.7	P_THB	16.4
26	W	HB	13.7	P_THB	14.4
25	W	HB	10.3	P_THB	11.0
36	M	HB	9.5	P_THB	10.2
79	W	HB	10.6	P_THB	11.3
86	W	HB	11.8	P_THB	12.5
85	M	HB	11.7	P_THB	12.4
25	W	HB	12.5	P_THB	13.2
69	W	HB	13.7	P_THB	14.4
42	M	HB	7.2	P_THB	7.9
74	M	HB	7.4	P_THB	8.1
82	W	HB	5.2	P_THB	5.9
84	M	HB	5.9	P_THB	5.2
66	W	HB	14.4	P_THB	15.1
45	W	HB	13.4	P_THB	14.1
78	M	HB	15.9	P_THB	16.6
57	M	HB	17.3	P_THB	18.0
83	M	HB	12.9	P_THB	13.6
67	M	HB	13.9	P_THB	14.6
51	M	HB	15.4	P_THB	16.1
56	M	HB	12.4	P_THB	13.1
18	M	HB	17.3	P_THB	18.0
70	M	HB	11.6	P_THB	10.9
23	W	HB	13.9	P_THB	14.6

71	W	HB	13.4	P_THB	14.1
57	M	HB	17.3	P_THB	18.0
88	W	HB	12.4	P_THB	13.1
26	M	HB	15.4	P_THB	16.1
50	M	HB	15.4	P_THB	16.1
26	M	HB	17.0	P_THB	17.7
94	W	HB	10.4	P_THB	11.1
19	M	HB	16.0	P_THB	16.7
70	M	HB	14.1	P_THB	13.4
50	M	HB	12.9	P_THB	13.6
69	W	HB	13.9	P_THB	14.6
68	M	HB	10.9	P_THB	11.6
25	M	HB	14.4	P_THB	15.1
43	M	HB	14.9	P_THB	15.6
63	M	HB	15.5	P_THB	16.2
58	M	HB	15.5	P_THB	16.2
53	W	HB	16.4	P_THB	17.1
73	M	HB	15.9	P_THB	16.6
15	W	HB	12.6	P_THB	11.9
58	W	HB	17.0	P_THB	16.3
67	M	HB	17.3	P_THB	18.0
74	M	HB	13.4	P_THB	14.1
38	M	HB	13.4	P_THB	14.1
67	M	HB	17.0	P_THB	17.7
52	M	HB	15.4	P_THB	16.1
38	M	HB	16.9	P_THB	17.6
80	W	HB	11.9	P_THB	12.6
53	W	HB	11.4	P_THB	12.1
66	M	HB	10.4	P_THB	11.1
33	W	HB	12.9	P_THB	13.6
32	M	HB	18.5	P_THB	19.2
78	W	HB	11.4	P_THB	12.1
48	W	HB	12.4	P_THB	13.1
20	W	HB	12.4	P_THB	13.1
53	M	HB	15.4	P_THB	16.1
71	W	HB	12.9	P_THB	13.6
34	W	HB	12.9	P_THB	13.6
70	M	HB	12.9	P_THB	13.6
33	W	HB	12.4	P_THB	13.1
27	W	HB	12.4	P_THB	13.1
48	M	HB	9.9	P_THB	10.6
18	W	HB	12.9	P_THB	13.6
48	W	HB	16.0	P_THB	16.7
49	W	HB	12.4	P_THB	13.1
48	W	HB	12.4	P_THB	13.1
34	M	HB	14.4	P_THB	15.1
73	M	HB	15.4	P_THB	16.1
78	W	HB	13.9	P_THB	14.6
25	M	HB	15.4	P_THB	16.1
67	M	HB	13.9	P_THB	14.6
47	W	HB	12.4	P_THB	13.1
74	W	HB	10.4	P_THB	11.1
24	M	HB	16.4	P_THB	17.1
44	M	HB	14.9	P_THB	15.6
25	W	HB	11.9	P_THB	12.6
72	M	HB	12.4	P_THB	13.1

20	W	HB	10.9	P_THB	11.6
21	M	HB	16.9	P_THB	17.6
88	W	HB	13.4	P_THB	14.1
64	M	HB	13.9	P_THB	14.6
22	M	HB	17.3	P_THB	18.0
62	M	HB	14.9	P_THB	15.6
79	M	HB	14.4	P_THB	15.1
29	M	HB	13.9	P_THB	14.6
56	M	HB	16.4	P_THB	17.1
83	W	HB	12.4	P_THB	13.1
39	W	HB	12.9	P_THB	13.6
42	M	HB	15.4	P_THB	16.1
50	W	HB	16.3	P_THB	17.0
74	M	HB	17.3	P_THB	18.0
31	W	HB	16.0	P_THB	16.7
65	M	HB	13.9	P_THB	14.6
52	M	HB	13.4	P_THB	14.1
15	M	HB	14.4	P_THB	15.1
46	W	HB	13.4	P_THB	14.1
45	M	HB	13.6	P_THB	12.9
77	W	HB	14.4	P_THB	15.1
77	W	HB	9.9	P_THB	10.6
68	M	HB	14.4	P_THB	15.1
78	M	HB	13.6	P_THB	14.4
68	M	HB	13.9	P_THB	13.1
36	M	HB	15.1	P_THB	15.9
29	M	HB	17.0	P_THB	17.8
59	W	HB	14.6	P_THB	15.4
33	M	HB	14.1	P_THB	14.9
26	M	HB	14.6	P_THB	15.4
75	M	HB	13.1	P_THB	13.9
56	W	HB	13.1	P_THB	13.9
18	W	HB	13.1	P_THB	13.9
32	M	HB	15.1	P_THB	15.9
46	W	HB	14.1	P_THB	14.9
48	W	HB	13.1	P_THB	13.9
37	M	HB	16.6	P_THB	17.4
19	M	HB	15.5	P_THB	16.3
36	M	HB	16.5	P_THB	17.3
65	M	HB	15.5	P_THB	16.3
53	M	HB	14.6	P_THB	15.4
58	M	HB	14.1	P_THB	14.9
43	W	HB	11.1	P_THB	11.9
95	W	HB	11.6	P_THB	12.4
26	M	HB	14.1	P_THB	14.9
54	M	HB	15.1	P_THB	15.9
74	W	HB	12.1	P_THB	12.9
25	W	HB	10.6	P_THB	11.4
39	W	HB	12.1	P_THB	12.9
28	M	HB	11.6	P_THB	12.4
27	M	HB	17.2	P_THB	18.0
34	M	HB	12.6	P_THB	13.4
77	W	HB	13.1	P_THB	13.9
67	M	HB	16.2	P_THB	17.0
57	W	HB	13.1	P_THB	13.9
31	M	HB	15.5	P_THB	16.3

19	M	HB	13.6	P_THB	14.4
30	M	HB	16.1	P_THB	16.9
36	M	HB	16.5	P_THB	17.3
63	M	HB	8.6	P_THB	9.4
82	W	HB	8.6	P_THB	9.4
52	M	HB	13.1	P_THB	13.9
65	M	HB	14.1	P_THB	14.9
71	M	HB	15.5	P_THB	16.3
50	M	HB	15.6	P_THB	16.4
33	W	HB	14.6	P_THB	15.4
78	W	HB	12.8	P_THB	13.6
25	W	HB	12.5	P_THB	13.3
53	W	HB	13.4	P_THB	14.2
36	M	HB	14.3	P_THB	15.1
53	M	HB	14.6	P_THB	13.8
23	W	HB	12.5	P_THB	13.3
80	W	HB	14.3	P_THB	15.1
70	W	HB	13.2	P_THB	14.0
23	W	HB	14.2	P_THB	15.0
24	W	HB	13.2	P_THB	14.0
69	W	HB	13.0	P_THB	13.8
83	W	HB	8.2	P_THB	9.0
84	M	HB	12.9	P_THB	13.7
78	M	HB	15.6	P_THB	14.8
67	M	HB	10.8	P_THB	11.6
86	W	HB	10.5	P_THB	11.3
25	W	HB	13.2	P_THB	14.0
36	W	HB	8.9	P_THB	9.7
47	M	HB	15.0	P_THB	15.8
80	M	HB	13.0	P_THB	13.8
77	M	HB	10.2	P_THB	11.0
79	W	HB	12.5	P_THB	13.3
21	W	HB	11.2	P_THB	12.0
88	W	HB	14.3	P_THB	15.1
80	W	HB	13.7	P_THB	14.5
45	W	HB	14.0	P_THB	13.2
24	M	HB	14.8	P_THB	15.6
22	W	HB	14.4	P_THB	15.2
34	M	HB	14.0	P_THB	14.8
23	M	HB	15.8	P_THB	16.6
20	W	HB	13.0	P_THB	13.8
51	W	HB	8.1	P_THB	7.3
31	M	HB	13.4	P_THB	14.2
80	W	HB	10.7	P_THB	11.5
39	W	HB	10.5	P_THB	11.3
22	W	HB	11.3	P_THB	12.1
33	M	HB	15.8	P_THB	16.6
32	W	HB	13.0	P_THB	13.8
72	M	HB	10.7	P_THB	11.5
75	W	HB	14.4	P_THB	15.2
79	M	HB	15.3	P_THB	16.1
62	M	HB	14.9	P_THB	15.7
57	M	HB	13.9	P_THB	14.7
29	M	HB	14.4	P_THB	15.2
35	M	HB	9.8	P_THB	10.6
87	M	HB	10.9	P_THB	11.7

87	W	HB	14.7	P_THB	15.5
83	W	HB	13.0	P_THB	13.8
75	W	HB	10.8	P_THB	11.6
88	W	HB	10.3	P_THB	11.1
56	W	HB	12.4	P_THB	13.2
45	M	HB	13.3	P_THB	14.1
76	M	HB	14.8	P_THB	15.6
25	W	HB	13.0	P_THB	13.8
20	M	HB	13.5	P_THB	14.3
79	W	HB	9.0	P_THB	9.8
59	W	HB	11.3	P_THB	12.1
70	M	HB	15.3	P_THB	16.1
76	W	HB	13.5	P_THB	14.3
85	M	HB	14.8	P_THB	15.6
71	W	HB	13.0	P_THB	13.8
83	W	HB	10.9	P_THB	11.7
37	M	HB	15.0	P_THB	15.8
54	W	HB	13.7	P_THB	14.5
45	M	HB	13.8	P_THB	13.0
54	M	HB	15.3	P_THB	16.1
51	M	HB	14.8	P_THB	15.6
56	W	HB	9.3	P_THB	10.1
51	M	HB	13.9	P_THB	14.7
69	M	HB	13.7	P_THB	14.5
42	M	HB	10.0	P_THB	10.8
13	M	HB	14.3	P_THB	15.1
43	M	HB	13.2	P_THB	14.0
24	M	HB	12.7	P_THB	13.5
57	M	HB	15.7	P_THB	16.5
60	M	HB	15.7	P_THB	16.5
47	M	HB	13.7	P_THB	14.5
29	W	HB	13.3	P_THB	14.1
74	M	HB	13.0	P_THB	13.8
73	M	HB	10.4	P_THB	11.2
25	W	HB	13.0	P_THB	13.8
37	W	HB	14.3	P_THB	15.1
78	M	HB	13.3	P_THB	12.5
38	M	HB	15.7	P_THB	16.5
50	M	HB	14.4	P_THB	15.2
23	W	HB	11.8	P_THB	12.6
69	M	HB	12.2	P_THB	13.0
48	M	HB	15.3	P_THB	16.1
35	W	HB	14.3	P_THB	15.1
58	W	HB	14.8	P_THB	15.6
19	W	HB	13.8	P_THB	14.6
30	M	HB	9.9	P_THB	10.7
54	W	HB	12.3	P_THB	13.1
35	W	HB	15.2	P_THB	16.0
36	W	HB	13.5	P_THB	14.3
83	W	HB	13.7	P_THB	14.5
65	M	HB	9.4	P_THB	10.2
45	M	HB	15.3	P_THB	16.1
62	M	HB	10.3	P_THB	11.1
22	M	HB	13.8	P_THB	14.6
82	M	HB	10.7	P_THB	11.5
67	W	HB	15.7	P_THB	16.5

87	W	HB	14.9	P_THB	15.7
41	M	HB	14.0	P_THB	14.8
17	M	HB	13.3	P_THB	14.1
70	W	HB	12.8	P_THB	13.6
45	W	HB	13.4	P_THB	14.2
81	M	HB	12.2	P_THB	13.0
48	W	HB	12.9	P_THB	13.7
33	M	HB	14.5	P_THB	15.3
61	W	HB	14.4	P_THB	15.2
51	W	HB	13.5	P_THB	14.3
70	M	HB	9.0	P_THB	9.8
62	W	HB	12.8	P_THB	13.6
31	W	HB	13.7	P_THB	14.5
77	W	HB	10.3	P_THB	11.1
75	M	HB	10.8	P_THB	11.6
55	W	HB	11.9	P_THB	12.7
57	M	HB	14.4	P_THB	15.2
79	M	HB	14.5	P_THB	15.3
70	M	HB	15.8	P_THB	16.6
22	W	HB	12.3	P_THB	13.1
58	M	HB	15.3	P_THB	16.1
35	M	HB	15.8	P_THB	16.6
18	M	HB	12.7	P_THB	13.5
31	W	HB	13.8	P_THB	14.6
59	W	HB	13.0	P_THB	13.8
27	M	HB	15.2	P_THB	16.0
68	W	HB	10.9	P_THB	11.7
63	M	HB	13.8	P_THB	14.6
85	M	HB	13.3	P_THB	14.1
57	M	HB	11.4	P_THB	12.2
28	M	HB	15.3	P_THB	16.1
70	W	HB	12.9	P_THB	13.7
77	W	HB	9.2	P_THB	10.0
48	M	HB	15.0	P_THB	15.8
58	W	HB	13.0	P_THB	13.8
76	W	HB	8.9	P_THB	9.7
38	M	HB	14.8	P_THB	15.6
40	W	HB	14.8	P_THB	15.6
52	M	HB	14.8	P_THB	15.6
21	W	HB	13.0	P_THB	13.8
50	W	HB	12.3	P_THB	13.1
22	M	HB	14.5	P_THB	15.3
41	M	HB	13.2	P_THB	12.4
94	W	HB	11.9	P_THB	12.7
46	M	HB	15.3	P_THB	16.1
34	W	HB	10.2	P_THB	11.0
24	M	HB	12.9	P_THB	13.7
50	M	HB	10.2	P_THB	11.0
32	W	HB	13.2	P_THB	14.0
86	M	HB	13.5	P_THB	14.3
52	M	HB	15.3	P_THB	16.1
74	M	HB	12.0	P_THB	12.8
53	M	HB	14.9	P_THB	15.7
67	M	HB	10.7	P_THB	11.5
23	W	HB	13.3	P_THB	14.1
45	M	HB	17.4	P_THB	18.2

47	M	HB	15.9	P_THB	16.7
31	M	HB	15.4	P_THB	16.2
25	M	HB	16.3	P_THB	17.1
71	M	HB	17.9	P_THB	18.7
40	M	HB	16.8	P_THB	17.6
62	M	HB	16.8	P_THB	17.6
47	M	HB	16.3	P_THB	17.1
47	M	HB	16.4	P_THB	17.2
34	M	HB	17.2	P_THB	18.1
58	W	HB	12.5	P_THB	13.4
74	M	HB	13.9	P_THB	13.0
65	W	HB	11.0	P_THB	11.9
84	M	HB	12.1	P_THB	13.0
40	W	HB	13.1	P_THB	14.0
15	W	HB	13.1	P_THB	14.0
88	W	HB	11.1	P_THB	12.0
47	M	HB	16.2	P_THB	17.1
44	W	HB	9.6	P_THB	10.5
63	W	HB	12.1	P_THB	13.0
55	M	HB	13.0	P_THB	13.9
66	M	HB	14.6	P_THB	15.5
34	M	HB	17.1	P_THB	18.0
66	W	HB	9.5	P_THB	10.4
44	M	HB	13.8	P_THB	14.7
57	W	HB	14.5	P_THB	15.4
21	W	HB	12.6	P_THB	13.5
40	M	HB	15.4	P_THB	16.3
23	M	HB	16.9	P_THB	17.8
49	M	HB	14.0	P_THB	14.9
25	M	HB	16.1	P_THB	17.0
48	M	HB	14.3	P_THB	15.2
47	M	HB	15.5	P_THB	16.4
84	W	HB	14.0	P_THB	14.9
37	M	HB	16.7	P_THB	17.6
40	W	HB	13.5	P_THB	14.4
34	W	HB	11.5	P_THB	12.4
40	W	HB	12.0	P_THB	12.9
67	M	HB	13.6	P_THB	14.5
76	W	HB	12.8	P_THB	13.7
71	W	HB	9.3	P_THB	10.2
58	W	HB	14.8	P_THB	15.7
32	W	HB	13.1	P_THB	14.0
65	M	HB	18.1	P_THB	19.0
52	M	HB	10.1	P_THB	11.0
55	M	HB	12.8	P_THB	13.7
57	W	HB	12.0	P_THB	12.9
63	M	HB	15.4	P_THB	16.3
56	M	HB	15.5	P_THB	16.4
82	W	HB	12.5	P_THB	13.4
75	W	HB	12.6	P_THB	13.5
72	W	HB	13.9	P_THB	13.0
70	M	HB	9.1	P_THB	10.0
22	W	HB	14.1	P_THB	15.0
51	W	HB	15.6	P_THB	16.5
31	W	HB	13.1	P_THB	14.0
46	W	HB	12.1	P_THB	13.0

31	W	HB	13.5	P_THB	14.4
32	M	HB	17.9	P_THB	18.8
58	M	HB	16.7	P_THB	17.6
34	M	HB	15.6	P_THB	16.5
50	W	HB	12.7	P_THB	11.8
18	W	HB	11.5	P_THB	12.4
51	M	HB	16.6	P_THB	17.5
39	M	HB	15.4	P_THB	16.3
41	W	HB	13.8	P_THB	14.7
27	W	HB	15.0	P_THB	15.9
16	M	HB	15.6	P_THB	16.5
87	W	HB	10.5	P_THB	11.4
60	W	HB	11.0	P_THB	11.9
27	M	HB	13.3	P_THB	14.2
76	W	HB	13.1	P_THB	14.0
51	W	HB	11.8	P_THB	12.7
50	W	HB	13.3	P_THB	14.2
56	M	HB	16.0	P_THB	16.9
74	W	HB	10.5	P_THB	9.6
28	W	HB	10.3	P_THB	11.2
42	M	HB	15.4	P_THB	16.3
17	W	HB	9.6	P_THB	10.5
17	W	HB	13.0	P_THB	13.9
75	M	HB	12.0	P_THB	12.9
53	W	HB	12.3	P_THB	13.2
52	M	HB	9.8	P_THB	10.7
31	M	HB	13.7	P_THB	14.6
54	M	HB	15.7	P_THB	16.6
75	W	HB	9.4	P_THB	10.3
74	W	HB	10.7	P_THB	11.6
59	M	HB	7.7	P_THB	8.6
52	M	HB	15.2	P_THB	16.1
26	W	HB	14.4	P_THB	15.3
65	M	HB	14.7	P_THB	15.6
23	W	HB	13.2	P_THB	14.1
19	W	HB	13.2	P_THB	14.1
80	W	HB	12.2	P_THB	13.1
59	M	HB	13.7	P_THB	14.6
23	M	HB	15.8	P_THB	16.7
71	W	HB	10.9	P_THB	11.8
77	W	HB	12.7	P_THB	13.6
43	M	HB	14.4	P_THB	15.3
77	W	HB	13.9	P_THB	14.8
26	W	HB	14.7	P_THB	15.6
44	W	HB	13.2	P_THB	14.1
80	W	HB	15.8	P_THB	16.7
81	M	HB	10.4	P_THB	11.3
40	W	HB	12.6	P_THB	11.7
77	M	HB	14.7	P_THB	15.6
90	M	HB	11.7	P_THB	12.6
32	W	HB	10.4	P_THB	11.3
95	M	HB	10.7	P_THB	11.6
90	W	HB	10.8	P_THB	9.9
49	W	HB	13.7	P_THB	14.6
56	M	HB	14.9	P_THB	15.8
78	M	HB	9.2	P_THB	10.1

75	M	HB	10.7	P_THB	11.6
64	W	HB	12.4	P_THB	13.3
10	M	HB	11.7	P_THB	12.6
44	W	HB	15.3	P_THB	16.2
38	M	HB	13.9	P_THB	14.8
63	M	HB	9.9	P_THB	10.8
78	W	HB	12.2	P_THB	13.1
45	M	HB	10.4	P_THB	11.3
57	M	HB	10.9	P_THB	11.8
52	M	HB	15.2	P_THB	16.1
45	W	HB	14.2	P_THB	15.1
28	W	HB	12.7	P_THB	13.6
79	M	HB	15.2	P_THB	16.1
72	M	HB	11.9	P_THB	12.8
80	W	HB	12.2	P_THB	13.1
81	M	HB	11.4	P_THB	12.3
80	W	HB	9.9	P_THB	10.8
71	M	HB	12.2	P_THB	13.1
45	W	HB	15.8	P_THB	16.7
50	M	HB	16.3	P_THB	17.2
55	M	HB	18.8	P_THB	19.7
44	M	HB	16.8	P_THB	17.7
62	M	HB	16.3	P_THB	17.2
65	M	HB	10.5	P_THB	11.5
55	W	HB	12.0	P_THB	13.0
24	W	HB	12.7	P_THB	13.7
80	M	HB	13.9	P_THB	14.9
76	M	HB	9.1	P_THB	10.1
20	M	HB	13.8	P_THB	14.8
64	M	HB	14.6	P_THB	15.6
38	M	HB	16.8	P_THB	17.8
25	W	HB	15.5	P_THB	16.5
19	M	HB	11.2	P_THB	12.2
30	M	HB	16.4	P_THB	17.4
83	M	HB	13.9	P_THB	14.9
76	W	HB	10.5	P_THB	11.5
88	W	HB	12.8	P_THB	13.8
34	W	HB	12.6	P_THB	13.6
23	W	HB	12.6	P_THB	13.6
19	M	HB	16.3	P_THB	17.3
59	M	HB	15.5	P_THB	16.5
82	W	HB	10.4	P_THB	11.4
79	M	HB	11.8	P_THB	12.8
46	M	HB	14.9	P_THB	15.9
53	W	HB	14.1	P_THB	15.1
56	W	HB	13.7	P_THB	14.7
89	W	HB	13.6	P_THB	14.6
63	M	HB	11.5	P_THB	12.5
70	M	HB	15.4	P_THB	16.4
76	M	HB	12.1	P_THB	13.1
72	W	HB	9.5	P_THB	10.5
85	M	HB	13.1	P_THB	14.1
29	W	HB	14.1	P_THB	15.1
79	W	HB	11.4	P_THB	12.4
20	W	HB	12.2	P_THB	13.2
19	W	HB	12.5	P_THB	13.5

73	W	HB	14.5	P_THB	15.5
50	W	HB	13.8	P_THB	14.8
23	M	HB	16.4	P_THB	17.4
90	W	HB	11.3	P_THB	12.3
73	W	HB	11.5	P_THB	12.5
56	M	HB	12.0	P_THB	13.0
28	W	HB	13.5	P_THB	14.5
73	M	HB	10.3	P_THB	11.3
31	M	HB	13.3	P_THB	14.3
36	W	HB	14.8	P_THB	15.8
25	M	HB	14.4	P_THB	13.4
44	W	HB	14.3	P_THB	15.3
26	W	HB	12.3	P_THB	13.3
35	W	HB	13.8	P_THB	12.8
65	W	HB	13.7	P_THB	14.7
17	W	HB	12.3	P_THB	13.3
66	M	HB	13.1	P_THB	14.1
77	W	HB	13.6	P_THB	12.6
51	M	HB	15.5	P_THB	16.5
63	M	HB	7.0	P_THB	8.0
76	W	HB	13.0	P_THB	12.0
68	M	HB	12.4	P_THB	13.4
92	W	HB	13.8	P_THB	14.8
29	M	HB	16.3	P_THB	17.3
18	W	HB	11.3	P_THB	12.3
17	W	HB	13.5	P_THB	14.5
73	W	HB	13.2	P_THB	14.2
75	M	HB	9.0	P_THB	10.0
63	W	HB	9.3	P_THB	10.3
36	M	HB	16.9	P_THB	17.9
53	M	HB	10.8	P_THB	9.8
54	W	HB	16.0	P_THB	17.0
62	W	HB	14.7	P_THB	13.7
62	M	HB	13.8	P_THB	12.8
63	M	HB	15.2	P_THB	14.2
76	W	HB	11.3	P_THB	12.3
61	W	HB	14.1	P_THB	15.1
76	W	HB	12.3	P_THB	13.3
62	W	HB	10.7	P_THB	11.7
58	M	HB	16.9	P_THB	17.9
71	M	HB	15.2	P_THB	16.2
67	M	HB	15.7	P_THB	16.7
23	W	HB	15.2	P_THB	16.2
79	M	HB	17.7	P_THB	18.8
74	W	HB	10.1	P_THB	11.2
60	W	HB	12.1	P_THB	13.2
56	M	HB	15.2	P_THB	16.3
55	W	HB	11.8	P_THB	12.9
76	W	HB	12.8	P_THB	13.9
76	M	HB	12.6	P_THB	13.7
37	W	HB	11.6	P_THB	12.7
43	M	HB	15.4	P_THB	14.3
75	M	HB	7.3	P_THB	8.4
77	M	HB	10.6	P_THB	11.7
79	M	HB	14.8	P_THB	15.9
21	W	HB	12.3	P_THB	13.4

89	W	HB	15.2	P_THB	16.3
74	M	HB	13.3	P_THB	14.4
74	W	HB	7.4	P_THB	8.5
70	W	HB	7.1	P_THB	6.0
55	W	HB	14.0	P_THB	15.1
49	M	HB	14.4	P_THB	15.5
42	M	HB	14.7	P_THB	15.8
44	M	HB	17.4	P_THB	18.5
57	W	HB	14.5	P_THB	15.6
24	W	HB	12.2	P_THB	13.3
52	W	HB	13.5	P_THB	14.6
74	W	HB	12.9	P_THB	14.0
28	M	HB	15.5	P_THB	14.4
29	W	HB	12.0	P_THB	10.9
51	W	HB	15.3	P_THB	14.2
70	M	HB	10.4	P_THB	11.5
79	W	HB	10.5	P_THB	11.6
81	M	HB	13.0	P_THB	11.9
22	M	HB	15.6	P_THB	14.5
75	W	HB	12.4	P_THB	13.5
63	W	HB	11.0	P_THB	12.1
30	M	HB	17.1	P_THB	18.2
82	W	HB	16.0	P_THB	17.1
41	W	HB	13.4	P_THB	14.5
31	M	HB	16.3	P_THB	17.4
70	M	HB	12.7	P_THB	13.9
37	W	HB	13.6	P_THB	14.8
61	W	HB	5.4	P_THB	6.6
60	W	HB	15.3	P_THB	16.5
84	M	HB	11.8	P_THB	13.0
48	W	HB	10.8	P_THB	12.0
32	M	HB	14.3	P_THB	15.5
34	M	HB	15.7	P_THB	16.9
38	W	HB	10.7	P_THB	11.9
50	M	HB	15.8	P_THB	17.0
75	M	HB	14.0	P_THB	12.8
74	W	HB	13.7	P_THB	14.9
78	M	HB	14.0	P_THB	15.2
70	M	HB	11.5	P_THB	12.7
66	W	HB	7.4	P_THB	8.6
83	M	HB	6.7	P_THB	7.9
77	W	HB	6.6	P_THB	7.8
44	M	HB	14.9	P_THB	16.1
51	M	HB	18.3	P_THB	19.5
49	M	HB	14.4	P_THB	15.6
84	W	HB	11.4	P_THB	12.6
37	W	HB	14.6	P_THB	13.4
73	M	HB	8.4	P_THB	9.6
61	M	HB	16.5	P_THB	17.7
51	M	HB	11.1	P_THB	12.4
55	M	HB	15.0	P_THB	16.3
19	W	HB	10.9	P_THB	12.2
73	W	HB	13.2	P_THB	11.9
52	W	HB	11.8	P_THB	10.5
28	M	HB	14.3	P_THB	15.6
25	M	HB	14.8	P_THB	16.1

89	M	HB	12.7	P_THB	14.0
49	W	HB	14.0	P_THB	15.3
49	M	HB	15.3	P_THB	16.6
86	W	HB	16.5	P_THB	15.1
90	M	HB	12.1	P_THB	13.5
26	W	HB	13.5	P_THB	14.9
64	M	HB	12.5	P_THB	11.1
86	M	HB	10.7	P_THB	9.3
53	W	HB	3.7	P_THB	2.3
16	M	HB	8.1	P_THB	6.7
50	W	HB	14.2	P_THB	15.6
75	M	HB	14.3	P_THB	12.9
61	M	HB	8.1	P_THB	6.7
59	W	HB	10.2	P_THB	11.6
80	W	HB	16.8	P_THB	18.2
31	M	HB	15.4	P_THB	16.9
63	M	HB	5.6	P_THB	7.1
65	W	HB	13.5	P_THB	12.0
82	W	HB	8.7	P_THB	7.2
56	W	HB	11.3	P_THB	12.8
16	M	HB	14.0	P_THB	15.5
71	M	HB	13.9	P_THB	12.4
46	W	HB	8.8	P_THB	10.3
70	W	HB	10.5	P_THB	12.0
71	M	HB	14.9	P_THB	13.4
73	W	HB	11.7	P_THB	10.1
63	W	HB	16.3	P_THB	14.7
85	M	HB	10.7	P_THB	12.3
74	W	HB	11.3	P_THB	9.6
90	W	HB	13.1	P_THB	11.4
65	M	HB	12.4	P_THB	14.1
38	M	HB	15.1	P_THB	13.4
27	M	HB	5.8	P_THB	7.6
90	W	HB	11.5	P_THB	13.3
71	M	HB	13.3	P_THB	11.5
54	W	HB	13.8	P_THB	15.6
85	W	HB	12.0	P_THB	13.8
79	M	HB	5.7	P_THB	7.5
50	M	HB	13.3	P_THB	15.1
77	M	HB	10.0	P_THB	11.8
58	M	HB	11.6	P_THB	13.5
62	M	HB	8.5	P_THB	10.4
85	W	HB	10.8	P_THB	12.7
57	W	HB	12.8	P_THB	14.7
79	W	HB	13.3	P_THB	15.2
46	W	HB	15.1	P_THB	13.1
23	M	HB	10.7	P_THB	8.6
59	W	HB	12.6	P_THB	10.5
67	M	HB	8.2	P_THB	10.3
56	M	HB	15.9	P_THB	13.7
25	W	HB	10.4	P_THB	8.2
94	W	HB	7.7	P_THB	5.5
77	W	HB	13.6	P_THB	11.4
47	W	HB	12.8	P_THB	10.5
50	W	HB	13.0	P_THB	15.3
68	W	HB	12.6	P_THB	10.3

32	M	HB	14.4	P_THB	12.0
25	M	HB	16.8	P_THB	19.2
77	W	HB	12.8	P_THB	15.3
59	M	HB	8.8	P_THB	11.4
40	M	HB	17.8	P_THB	15.1
80	W	HB	10.5	P_THB	13.2
32	W	HB	15.1	P_THB	12.4
75	W	HB	12.7	P_THB	9.8
66	M	HB	13.7	P_THB	10.8
58	W	HB	13.0	P_THB	10.1
35	M	HB	15.9	P_THB	19.0
67	W	HB	10.1	P_THB	13.3
85	W	HB	15.5	P_THB	12.3
54	W	HB	14.1	P_THB	10.8
34	M	HB	15.6	P_THB	12.1
58	W	HB	14.5	P_THB	18.0
73	M	HB	14.9	P_THB	11.1
73	M	HB	7.2	P_THB	11.1
55	W	HB	9.7	P_THB	13.7
76	M	HB	10.3	P_THB	14.4
40	M	HB	8.9	P_THB	13.0
46	M	HB	8.2	P_THB	12.3
65	W	HB	5.7	P_THB	10.1
62	W	HB	2.6	P_THB	7.1
83	W	HB	8.2	P_THB	13.1
30	W	HB	9.7	P_THB	15.0
72	W	HB	9.8	P_THB	3.9
31	W	HB	14.2	P_THB	20.2
48	M	HB	7.9	P_THB	15.1
